# A Species-Specific Cluster of Defensin-Like Genes Encodes Diffusible Pollen Tube Attractants in *Arabidopsis*

**DOI:** 10.1371/journal.pbio.1001449

**Published:** 2012-12-18

**Authors:** Hidenori Takeuchi, Tetsuya Higashiyama

**Affiliations:** 1 Division of Biological Science, Graduate School of Science, Nagoya University, Nagoya, Aichi, Japan; 2 JST ERATO Higashiyama Live-Holonics Project, Nagoya University, Nagoya, Aichi, Japan; University of Zurich, Switzerland

## Abstract

Genes directly involved in male/female and host/parasite interactions are believed to be under positive selection. The flowering plant *Arabidopsis thaliana* has more than 300 defensin-like (*DEFL*) genes, which are likely to be involved in both natural immunity and cell-to-cell communication including pollen–pistil interactions. However, little is known of the relationship between the molecular evolution of *DEFL* genes and their functions. Here, we identified a recently evolved cluster of *DEFL* genes in *A. thaliana* and demonstrated that these DEFL (cysteine-rich peptide [CRP810_1]) peptides, named AtLURE1 peptides, are pollen tube attractants guiding pollen tubes to the ovular micropyle. The *AtLURE1* genes formed the sole species-specific cluster among *DEFL* genes compared to its close relative, *A. lyrata*. No evidence for positive selection was detected in *AtLURE1* genes and their orthologs, implying neutral evolution of *AtLURE1* genes. AtLURE1 peptides were specifically expressed in egg-accompanying synergid cells and secreted toward the funicular surface through the micropyle. Genetic analyses showed that gametophytic mutants defective in micropylar guidance (*myb98*, *magatama3*, and *central cell guidance*) do not express AtLURE1 peptides. Downregulation of the expression of these peptides impaired precise pollen tube attraction to the micropylar opening of some populations of ovules. Recombinant AtLURE1 peptides attracted *A. thaliana* pollen tubes at a higher frequency compared to *A. lyrata* pollen tubes, suggesting that these peptides are species-preferential attractants in micropylar guidance. In support of this idea, the heterologous expression of a single AtLURE1 peptide in the synergid cell of *Torenia fournieri* was sufficient to guide *A. thaliana* pollen tubes to the *T. fournieri* embryo sac and to permit entry into it. Our results suggest the unique evolution of *AtLURE1* genes, which are directly involved in male–female interaction among the *DEFL* multigene family, and furthermore suggest that these peptides are sufficient to overcome interspecific barriers in gametophytic attraction and penetration.

## Abstract 2

AtLURE1 defensin-like peptides, which show species-specific evolution, are essential in *Arabidopsis* for attracting pollen tubes and can function in the breakdown of reproductive isolation barriers.

## Author Summary

Defensin-like (DEFL) peptides commonly function as effector peptides and are involved in male-female and host-parasite interactions in eukaryotes. In higher plants, *DEFL* genes belong to a large multigene family and are highly variable between species. However, little is known about the relationship between the molecular evolution of *DEFL* genes and their functions. By comparing multiply duplicated *DEFL* genes between *A. thaliana* and its close relative *A. lyrata*, we have now identified pollen tube attractant peptides called AtLURE1 peptides, in *A. thaliana*. We find that *AtLURE1* genes form a species-specific gene cluster and that the AtLURE1 peptides these genes encode are specifically expressed in the synergid (egg-accompanying) cells and are secreted along the path down which the pollen tube elongates to reach the female gametophyte. AtLURE1 peptides attract pollen tubes in a species-preferential manner and their downregulation impairs pollen tube guidance. Interestingly, the genetic introduction of a single *AtLURE1* gene from *A. thaliana* into another plant, *T. fournieri*, is sufficient to breakdown reproductive isolation barriers in pollen tube guidance and penetration. These results suggest that AtLURE1 peptides, which show species-specific evolution, are key molecules that attract pollen tubes from a plant's own species to the embryo sac to enable successful reproduction.

## Introduction

Disease and sexual reproduction are believed to have profound effects on molecular evolution because host–parasite and male–female interactions exert selection pressures [1],[2]. Genes involved in these interactions tend to show characteristic molecular evolution under positive Darwinian selection [2]–[6] and rapid gene turnover (gain/loss) [7]–[9]. However, the relationship between the evolutionary pattern and function of each gene still remains unclear.

Defensins are antimicrobial peptides involved in innate immunity that are generally found in eukaryotes, including mammals, insects, and plants [10]. Plant defensins have a structure consisting of a triple-stranded β-sheet with an α-helix in parallel [11]. *Defensin-like* (*DEFL*) genes of flowering plants form a large multigene family, with 317 *DEFL* genes in *A. thaliana* (accession Col-0) [12] and 93 *DEFL* genes in *Oryza sativa* (rice) [13]. In *A. thaliana*, *DEFL* genes are subdivided into 46 subgroups by the number and alignment of cysteine residues [12]. They have evolved by tandem and segmental duplication events, and some individual subgroups include paralogous, multiply duplicated genes [12].

Plant DEFL peptides are involved not only in the innate immune system but also in various steps in male–female interactions in plant sexual reproduction [14]. For example, the SCR/SP11 peptide in Brassicaceae is the male determinant of self/nonself-recognition during pollination [15],[16]. LURE peptides of *T. fournieri* (Linderniaceae, Lamiales) are specifically expressed in the synergid cell (an egg-accompanying haploid cell) to attract pollen tubes [17]. The *Zea mays* EMBRYO SAC 4 (ZmES4) peptide of *Z. mays*, predominantly expressed in the synergid cell, induces pollen tube rupture for the release of the sperm cell [18]. Thus, plant *DEFL* genes are likely to form a unique multigene family directly involved in both defense and sexual reproduction. Genome-wide analysis of the 317 *DEFL* genes of *A. thaliana* suggested rapid molecular evolution, including local duplication, to form gene clusters [12],[13]. However, whether any characteristically evolving *DEFL* genes exist among the *DEFL* genes of *A. thaliana*, and whether such genes are actually involved in disease or sexual reproduction, are not known.

In this study, we surveyed the *DEFL* genes of *A. thaliana* showing unique molecular evolution (in the form of rapid gene turnover) using whole-genome data of a closely related species, *A. lyrata* [19]. We especially focused on paralogous genes because rapid gene turnover has sometimes been suggested in multigene families, including the antimicrobial peptides of *Drosophila* [8]. We discovered a sole species-specific *DEFL* gene cluster that was demonstrated to encode attractant peptides for pollen tubes in *A. thaliana*. In addition, the heterologous expression of an attractant peptide was sufficient to overcome reproductive isolation barriers in the final steps of male–female interactions despite a large evolutionary distance between organisms.

## Results

### Identification of a Species-Specific Cluster of *DEFL* Genes in *A. thaliana*

**Figure 1:**
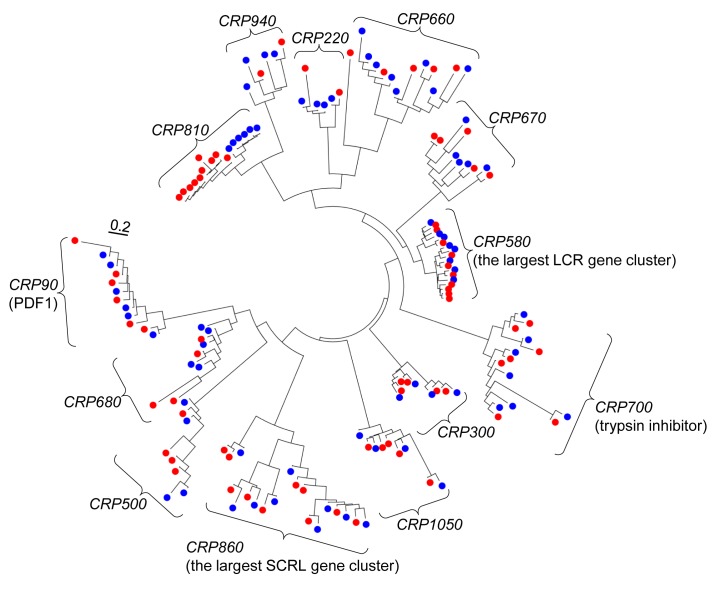
A phylogenetic tree of paralogous *DEFL* genes of *A. thaliana* and a close relative *A. lyrata*. The tree includes 13 subtrees, which belong to 13 *CRP* subgroups. Each subtree contains four or more paralogous *DEFL* genes of *A. thaliana* (blue filled circle) and their orthologs from *A. lyrata* (red filled circle). The scale shows the number of substitutions per site. The *CRP90* subtree contains *AtPDF1* genes [11]. The *CRP700* subtree contains *A. thaliana* trypsin inhibitor (*ATTI*) genes [75]. The *CRP580* and *CRP860* subtrees contain genes that form the largest gene cluster of *LCR* (low molecular weight, cysteine-rich) and *SCRL* (*SCR*-like), respectively [76]. The *CRP810* subtree branched into *A. thaliana* and *A. lyrata* gene clusters. Also see Table S1 for sequences of these *DEFL* genes.

To survey *DEFL* genes showing lineage-specific expansion, we first identified paralogous genes among the 317 *DEFL* genes of *A. thaliana* by a phylogenetic tree analysis using 317 putative DEFL peptide sequences [12]. We focused on four or more paralogous genes supported by high bootstrap values (≥90%) that could have evolved by recent multiple gene duplication, and found 13 groups of the multiple paralogous genes (Table S1). To investigate interspecific variation and the origin of these genes, we searched for orthologs in the close relative *A. lyrata* (accession CS22696) [19]. Multiple orthologs for the 13 groups of the *A. thaliana* genes were found in the *A. lyrata* genome by BLAST searches (Table S1). Notably, a phylogenetic tree analysis for the *A. thaliana* genes of the 13 groups and the *A. lyrata* orthologous genes showed that a single subtree, which includes six *DEFL* (*Cysteine-Rich Peptide 810*; *CRP810*) genes of *A. thaliana* and ten orthologs of *A. lyrata*, branched into species-specific gene clades for *A. thaliana* and *A. lyrata* (Figure 1). No other clades, including *CRP90* (true plant defensin *PDF1* genes [11]), showed such a phylogenetic relationship between *A. thaliana* and *A. lyrata* (Figure 1). This result suggested that these *CRP810* genes diverged after the split between *A. thaliana* and *A. lyrata* [20]. We decided to further analyze these *CRP810* genes, *CRP810_1.1* to _*1.6* and *AlCRP810_1.1* to *_1.10*, numbered according to their chromosomal locations (Table S1).

**Figure 2:**
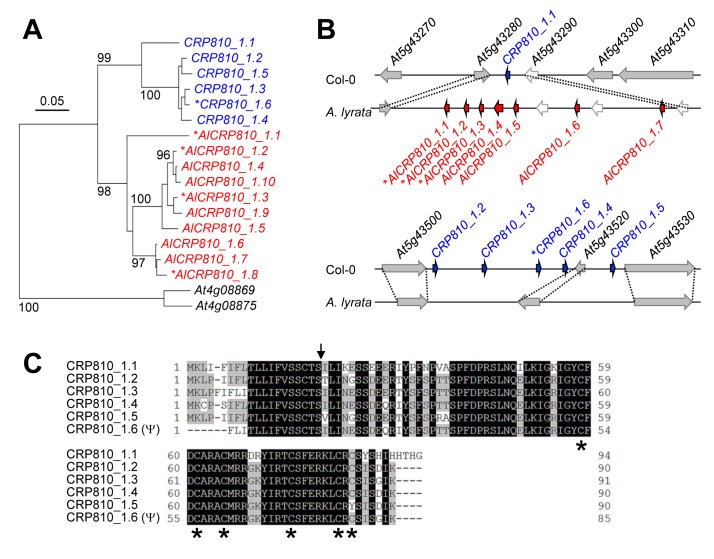
Phylogenetic and syntenic relationship between the *CRP810_1* genes of *A. thaliana* and *A. lyrata*. (A) Phylogenetic tree of the *CRP810_1* (blue) and *AlCRP810_1* (red) genes based on the coding region of their genomic sequences, with *At4g08869* and *At4g08875* (the closest related genes to *CRP810_1* genes) as the outgroup. Only bootstrap values ≥90 are indicated. The scale shows the number of substitutions per site. (B) Representations of syntenic regions containing *CRP810_1* and *AlCRP810_1* genes in the *A. thaliana* (Col-0) and *A. lyrata* genomes. Blue and red arrows represent *CRP810_1* and *AlCRP810_1* genes, respectively. Gray arrows show flanking genes. Dashed lines indicate syntenic genes between *A. thaliana* and *A. lyrata*. The genes with asterisks in (A and B) are probably nonfunctional. Also see Figure S1. (C) Full-length amino acid sequences of CRP810_1.1 to _1.5 and CRP810_1.6 (Ψ). The arrow indicates the position of the predicted cleavage sites. Asterisks mark conserved cysteine residues. Also see Figure S2 for comparison with AlCRP810_1.

To elucidate how these genes evolved, we performed phylogenetic and synteny analyses of the *CRP810_1* and *AlCRP810_1* genes. Consistent with the phylogenetic analysis showing that the *CRP810_1* and *AlCRP810_1* genes clustered independently to form phylogenetically distinct groups (Figures 1 and 2A), the synteny analysis indicated that a single gene (*CRP810_1.1*) only showed synteny with the *AlCRP810_1* gene (Figure 2B, top). No *AlCRP810_1* gene was present on the contig of *A. lyrata* showing synteny with the *CRP810_1.2*–*1.6* region (Figure 2B, bottom). In summary, in *A. thaliana*, an ancestral gene (*CRP810_1.1*) likely was copied to one of the loci of *CRP810_1.2*–*1.6* on the same chromosome and then duplicated locally (within 15 kb). In contrast to this, the other 12 groups of paralogous *DEFL* genes of *A. thaliana* and *A. lyrata* are not phylogenetically distinct groups (Figure S1A) and showed synteny with multiple genes (Figure S1B). We have thus identified the *CRP810_1* genes as the sole species-specific gene cluster among the 317 *DEFL* genes of *A. thaliana*.

### Characterization of CRP810_1 Peptides

*CRP810_1* consists of six paralogous genes, of which five are functional genes (*CRP810_1.1* to _*1.5*) and one is a pseudogene (*CRP810_1.6*), as a subset of the 16 *CRP810* genes [12]. They encode ∼90 amino acids with N-terminal secretory signal sequence, containing six cysteines in their putative mature peptides, with a CXC motif at the C-terminus (Figure 2C). *CRP810_1.1* to *_1.5* had 80%–95% amino acid sequence identity. *CRP810_1.6* had a 1-bp deletion at the 17th nucleotide compared to the sequences of other *CRP810_1* genes that induces a frameshift, resulting in a truncated, nonfunctional protein. The putative original amino acid sequence of CRP810_1.6 (Ψ), as deduced from nucleotides 18–275 of *CRP810_1.6*, also showed 80%–95% identity with CRP810_1.1 to _1.5. CRP810_1 peptides had the same number and alignment of cysteines and showed ∼70% amino acid sequence identity with the AlCRP810_1 peptides (Figure S2). A conserved cysteine residue (amino acid 84) in CRP810_1.5 was changed to a tyrosine.

### CRP810_1 Peptides Are Specifically Expressed in Synergid Cells and Secreted toward the Funicular Surface through the Micropyle

**Figure 3:**
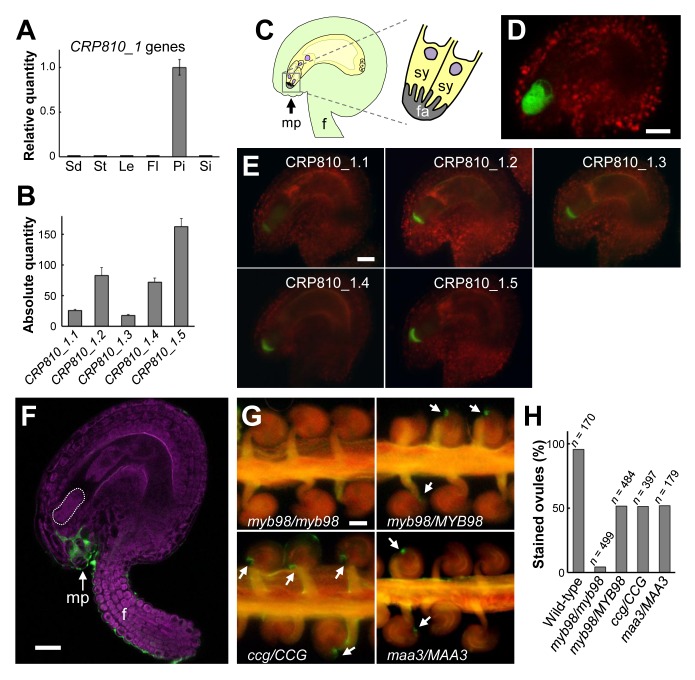
Expression pattern and localization of CRP810_1 peptides. (A) Real-time qRT-PCR analysis of *CRP810_1* genes (*CRP810_1.1* to _*1.6*) in seedlings 10 d after germination (Sd), stems (St), leaves (Le), open flowers without the pistil (Fl), pistils (Pi), and siliques 2 d after pollination (Si). Relative quantities are expression levels relative to that in the pistil. Each expression level was normalized to that of *ACT2*. The data are the means and standard errors of three independent samples. (B) Absolute gene expression levels of *CRP810_1* genes. Absolute quantity represents the copy number of cDNA per that of *MYB98* cDNA. The means and standard deviations of three independent experiments are shown. (C) Schematic of the ovule (left) and part of the synergid cell (sy) (right) in *A. thaliana*. The filiform apparatus (fa) is formed by the thickened cell walls of the synergid cells. f, funiculus; mp, micropyle. (D) Confocal laser scanning microscopic (CLSM) image of a *pCRP810_1.2::GFP* ovule. Scale bar, 20 µm. (E) Fluorescence microscopic images of *pCRP810_1::CRP810_1-GFP* ovules. Scale bar, 20 µm. (F) A CLSM image of an ovule after immunostaining with anti-CRP810_1.2 antibodies. Green Alexa Fluor fluorescence (for CRP810_1 peptides) was observed at the micropyle and funicular surface. Magenta indicates autofluorescence of the ovule. The synergid cell is delineated by the dashed line. Scale bar, 20 µm. Also see Figure S3. (G) Immunostaining with anti-CRP810_1.2 antibodies for the female gametophytic mutants *myb98*/*myb98*, *myb98*/*MYB98*, *ccg*/*CCG*, and *maa3*/*MAA3*. Representative fluorescence microscopic images of ovules on the septum are shown. Arrows indicate fluorescence around the micropylar end of the ovules. Scale bars, 50 µm. (H) The rate of immunostained ovules to the total number (*n*) of ovules in the wild type and mutants.

To determine the functions of CRP810_1 peptides, we investigated their expression patterns and localization. Quantitative reverse transcription (qRT)-PCR with vegetative and reproductive tissues revealed predominant *CRP810_1* expression in pistil tissue (Figure 3A). We also showed strong expression of *CRP810_1.1* to *_1.5* in the pistil (Figure 3B). Consistent with these results, previous data from a genome-wide analysis of *A. thaliana* ovules suggested that *CRP810_1* genes are expressed in the female gametophyte inside the pistil and downregulated in a *myb98* T-DNA insertion mutant [21]. The *MYB98* gene encodes a transcription factor predominantly expressed in the synergid cell and is necessary for pollen tube guidance at the micropyle of the ovule and development of the filiform apparatus of the synergid cell, a cell-wall ingrown structure [22],[23]. In addition, we confirmed by custom array analysis that they were upregulated in mature ovules compared with seedlings (unpublished data), suggesting that they might be specifically expressed in the female gametophyte. In ovules expressing green fluorescent protein (GFP) under the control of the promoters of *CRP810_1.1* to *_1.5*, GFP fluorescence was observed exclusively in the synergid cells (Figure 3D). We also generated transgenic plants that expressed GFP-fused CRP810_1 regulated by their own promoters. All GFP-fused CRP810_1 proteins were localized to the micropylar end of the female gametophyte (Figure 3E), possibly at the filiform apparatus of the synergid cell. This was consistent with the previous observation of GFP-fused CRP810_1.2 (DD2) [24]. These results indicate that CRP810_1 peptides are specifically expressed in the synergid cell and are likely secreted toward the micropylar end of the female gametophyte.

Immunostaining of the ovule using an anti-CRP810_1.2 antibody showed that the CRP810_1 peptides were secreted from the synergid cell toward the micropylar opening and then diffused to the funicular surface of the ovule (Figures 3F and S3A). The antibody recognized all of the CRP810_1 peptides in an immuno-dot blot analysis (Figure S3B). The GFP-fused CRP810_1 proteins did not show this diffuse pattern, most likely due to the increased molecular weight of the fusion protein (∼40 kDa), which is close to the transmission limit of the cell wall. Diffusion of the CRP810_1 peptides suggested the possibility that these peptides may be effective at a distance from the synergid cell. This raised the possibility that the CRP810_1 peptides may be involved in pollen tube guidance on the funiculus.

Accordingly, we next examined the relationship between the CRP810_1 peptides and known micropylar guidance mutants. *CRP810_1* genes have been suggested to be downregulated in a *myb98* mutant defective in micropylar guidance [21]. In addition to *myb98*, *central cell guidance* (*ccg*), a mutant defective in a nuclear protein expressed in the central cell [25], and *magatama3* (*maa3*), a mutant possibly defective in RNA metabolism in ubiquitous cells [26], were investigated as female gametophytic mutants defective in micropylar guidance. We performed immunostaining on *myb98/myb98* homozygous and *myb98*/*MYB98*, *ccg*/*CCG*, and *maa3*/*MAA3* heterozygous mutants (homozygous mutants for *ccg* and *maa3* were not obtained [25],[26]). Secreted CRP810_1 peptides were detected in only half of the ovules in the pistils of *myb98*/*MYB98* (51.7%, *n* = 484), *ccg*/*CCG* (51.4%, *n* = 397), and *maa3*/*MAA3* (52.0%, *n* = 179) mutants, in contrast with wild-type (95.9%, *n* = 170) and *myb98*/*myb98* (4.4%, *n* = 499) ovules (Figure 3G and 3H). Therefore, ovules lacking fluorescence in the heterozygous mutants were implicated as mutant ovules because the expected segregation ratio of wild-type to mutant female gametophytes was 50% in the heterozygous mutants. These results indicate that the synergid cells of these micropylar guidance mutants produce less CRP810_1 peptide and support the notion that the CRP810_1 peptides are involved in pollen tube attraction around or toward the micropyle.

### Diffusible CRP810_1 Peptides Are Involved in Micropylar Guidance

**Figure 4:**
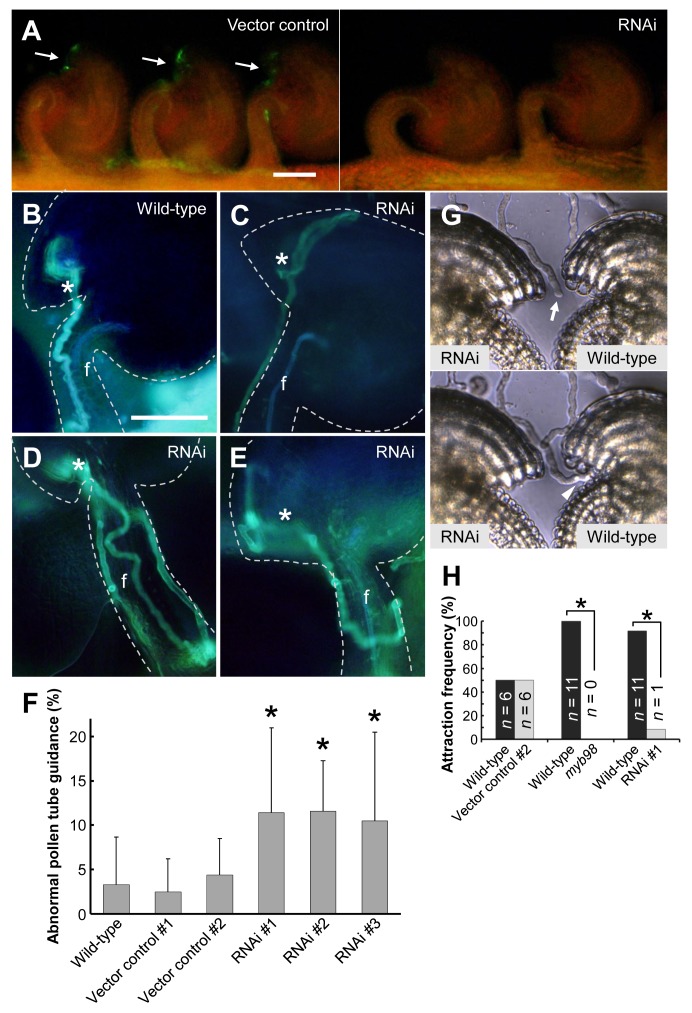
Knockdown analyses of the defects in micropylar guidance. (A) Representative fluorescence microscopic image of immunostained ovules on the septum with anti-CRP810_1.2 antibodies for the vector control and RNAi line. Arrows indicate fluorescence around the micropylar end of the ovules. Scale bar, 50 µm. (B–E) Aniline blue staining for pollen tubes inside the pistil. Asterisks indicate the micropylar opening. The ovule is delineated by the dashed line. f, funiculus. (B) The pollen tube was attracted normally to the wild-type ovule. (C) The pollen tube went past the micropylar opening and then turned back and grew into the micropyle. (D) The pollen tube growing on the funiculus (left side) went back down (right side) near the micropylar opening and then grew on the funiculus again (center) and reached the micropyle. (E) The wandering pollen tube failed to grow into the micropyle. Scale bar, 50 µm for (B–E). (F) Summary of abnormal pollen tube guidance around the micropylar opening in wild-type and transgenic ovules. Sums of class I and class II abnormalities in pollen tube guidance are shown. The data are averages and standard deviations of the frequencies per pistil. The total numbers of counted ovules are 339, 230, 183, 389, 135, and 173 for wild-type, vector control #1, #2, RNAi #1, #2, and #3 ovules, respectively. The frequencies of RNAi lines (#1–3) are significantly different from the wild type (asterisks, *p*<0.01). (G and H) In vitro comparative pollen tube attraction assay. (G) An arrow indicates the tip of the pollen tube that was initially between the wild-type and RNAi ovules (upper panel). The pollen tube was preferentially attracted to the wild-type ovule (lower panel). An arrowhead indicates the pollen tube entering the wild-type micropyle. (H) Summary of attraction frequencies in this assay. The attraction frequencies for the wild-type ovule (black boxes) or competing ovules (gray boxes) are represented collaterally. The numbers (*n*) are the total number of pollen tubes competitively attracted to the ovules. Asterisks indicate significant differences from a 1∶1 ratio in the binomial test (*p*<0.01). Also see Figure S4.

To define the function of CRP810_1 peptides *in vivo*, we simultaneously downregulated the five paralogous genes (*CRP810_1.1* to *_1.5*) using RNA interference (RNAi) against the complete *CRP810_1.2* coding sequence (Figure S4A). We obtained 11 independent T_1_ lines of *CRP810_1*-RNAi (RNAi), 14 lines of *MYB98*-RNAi as positive controls, and 13 lines of a linker for the RNAi construct (vector control). All 14 *MYB98*-RNAi T_1_ lines showed reduced fertility (44%±15%, mean%±SD for the rate of developing seeds upon selfing) and a defect in micropylar guidance similar to the *myb98* mutant (unpublished data); thus, our RNAi system appeared to work. We also confirmed the specific downregulation of all *CRP810_1* genes in *CRP810_1*-RNAi lines by qRT-PCR (Figure S4B). To analyze the RNAi lines in greater detail, we selected three T_3_ RNAi lines derived from three independent T_1_ lines in which CRP810_1 peptides were barely observed in the micropylar opening by immunostaining (Figure 4A).

Some disordered patterns in pollen tube growth were observed around the micropylar opening in these RNAi lines after aniline blue staining. To evaluate abnormal pollen tube guidance around the micropylar opening, we classified defects in micropylar guidance into two groups. The first included those cases in which one or more pollen tubes wandered around the micropyle but finally entered the micropyle (class I abnormality; Figure 4C and 4D); the second was more severe, in which no pollen tube entered the micropyle after growing up the funiculus (class II abnormality; Figure 4E). Approximately 10% of the ovules in each RNAi line showed class I and class II abnormalities (Figure 4F). Of these, ∼1% exhibited a class II abnormality, which was never observed in the ovules of the wild-type or vector control lines. The frequency of class I and class II abnormalities in all three RNAi lines was significant (Student's *t* test; *p*<0.01). Thus, we conclude that the downregulation of *CRP810_1* genes impaired micropylar pollen tube guidance, although it did not result in a complete loss of micropylar guidance. In the micropylar guidance mutants, the phenotype of abnormal pollen tube guidance was more severe and was observed at higher frequencies than in the RNAi lines; class I and class II abnormalities were 20%±14% and 65%±12% in *myb98/myb98*, 20%±12% and 14%±11% in *myb98/MYB98*, 22%±13% and 19%±10% in *ccg/CCG*, and 28%±15% and 17%±14% in *maa3/MAA3*, respectively.

To examine whether *CRP810_1* downregulation actually impaired the ability of the ovules to attract pollen tubes, we performed a competitive assay semi-in vitro using wild-type and RNAi ovules simultaneously (Figure 4G). We placed a wild-type and an RNAi ovule together facing the micropylar opening so that a pollen tube could grow between the ovules. Ovules of homozygous *myb98* (*myb98-1*) were also examined. The pollen tubes were rarely attracted to the RNAi ovules (8.3%, *n* = 12) and never to *myb98* ovules (0%, *n* = 11) (Figure 4H). These results indicate that the loss of CRP810_1 peptides impaired pollen tube attraction to the micropylar opening in vivo and in vitro.

### CRP810_1 Peptides Containing Six Cysteines Can Attract *A. thaliana* Pollen Tubes

**Figure 5:**
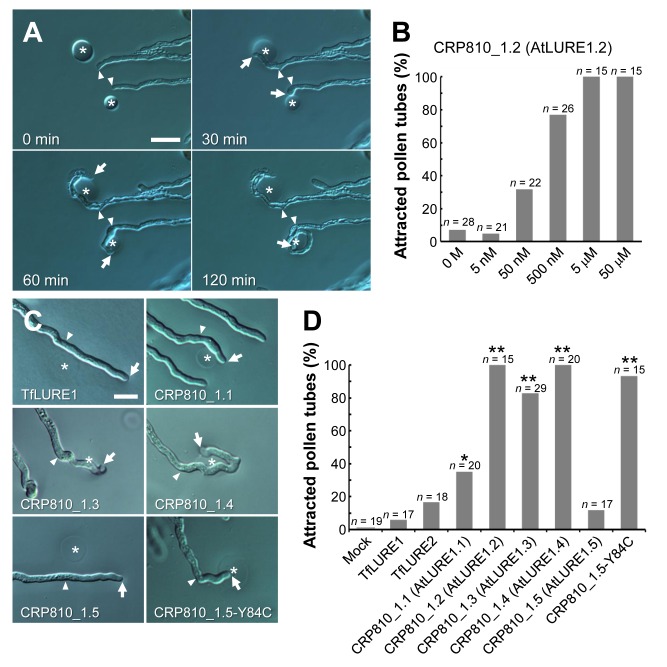
In vitro pollen tube attraction assay using recombinant proteins. (A) Pollen tube attraction toward gelatin beads containing 50 µM histidine-tagged CRP810_1.2. Arrowheads mark the position of the pollen tube tips when the gelatin beads (asterisk) were placed (0 min). Arrows indicate the tips of pollen tubes growing toward the beads 30 and 60 min after placement. At 60 min, the upper pollen tube was spontaneously disrupted and the lower pollen tube was trapped at the bead. Scale bar, 20 µm. (B) Concentration-dependent pollen tube attraction activity of CRP810_1.2 (AtLURE1.2). The data are the frequencies for the total number of pollen tubes (*n*) in at least three assays. Pollen tubes growing toward beads with a >30° change were designated as attracted pollen tubes. (C) Representative samples of attracted or non-attracted pollen tubes to recombinant TfLURE1 and CRP810_1. Arrowheads mark the position of the pollen tube tip when the gelatin beads (asterisk) were placed. Arrows indicate the tips of the pollen tubes. Scale bar, 20 µm. (D) Summary of the rates of attraction of the pollen tubes to each recombinant protein. The data are the frequencies for the total number of pollen tubes (*n*) in at least three assays per protein. An asterisk and double asterisks indicate significant differences compared with buffer alone (0 M) (Figure 5B) using Fisher exact test (*p<0.03; **p<0.01). Also see Figure S5.

CRP810_1 peptides expressed in the synergid cell were shown to diffuse toward the micropyle and were involved in pollen tube attraction at the micropyle. We next examined whether CRP810_1 peptides can directly attract pollen tubes. Recombinant His-tagged CRP810_1.1 to _1.5, following the putative cleavage site in the signal peptide, were individually expressed in *Escherichia coli*. These recombinant peptides were purified and refolded according to the procedures used for TfLUREs [17]. Pollen tubes were grown through a style on medium using an in vitro system (Figure S5A) [27],[28]. The purified peptides were embedded in gelatin beads ∼15 µm in diameter for micromanipulation (Figure S5B and S5C). First, we investigated the activity of CRP810_1.2 and its concentration dependency (Figure 5A and 5B), since the expression level of *CRP810_1.2* was the highest among *CRP810_1* genes, except for *CRP810_1.5*, which lacks a conserved cysteine residue. When gelatin beads containing 50 µM CRP810_1.2 were placed in front of the tip of a pollen tube, 100% (*n* = 15) of the tubes turned sharply toward the beads (Figure 5A). Some tubes formed narrow coils around the beads (Figure 5A). Beads containing 5 µM CRP810_1.2 also attracted all of the pollen tubes examined (*n* = 15). As the concentration of CRP810_1.2 in the beads decreased to 500 and 50 nM, the frequency of pollen tube attraction decreased (77%, *n* = 26 versus 32%, *n* = 22). Beads containing buffer alone (0 M) could not attract pollen tubes (Figure 5B; 7%, *n* = 28). This low frequency (7%) was likely due to random directional change of the pollen tube growth, which was also observed without beads. These results suggest that CRP810_1.2 is sufficient to attract pollen tubes in a concentration-dependent manner.

Next, we examined each recombinant peptide of CRP810_1.1, _1.3, _1.4, and _1.5 at a concentration of 50 µM (Figure 5C and 5D). Pollen tubes were attracted to CRP810_1.3 and _1.4 at a high frequency (83%, *n* = 29 and 100%, *n* = 20, respectively) and CRP810_1.1 at a lower frequency (35%, *n* = 20). In contrast, CRP810_1.5, lacking one of the conserved cysteines, did not attract pollen tubes (12%, *n* = 17). Note that mutated CRP810_1.5-Y84C, in which the 84th tyrosine reverted to a cysteine, showed significant activity (93%, *n* = 15). This suggests that a single amino acid substitution inhibited the pollen tube attraction activity of CRP810_1.5 and that cysteine residues and possibly the formation of disulfide bonds in CRP810_1 peptides are required for their activity. Furthermore, TfLURE1 and 2, which are attractants from *T. fournieri*, did not show significant attraction of *A. thaliana* pollen tubes (6%, *n* = 17 and 17%, *n* = 18, respectively) (Figure 5C and 5D). In our pollen tube attraction assay, pollen tubes were frequently trapped or deviated by more than a 90° angle toward beads containing CRP810_1 peptides, but they never deviated by more than a 45° angle toward beads containing buffer alone or TfLUREs, supporting the notion of specific attraction by CRP810_1 peptides. Since CRP810_1 peptides containing six cysteines, including CRP810_1.5-Y84C, had the ability to attract pollen tubes, we named the CRP810_1 peptides AtLURE1 peptides (e.g., CRP810_1.1 is AtLURE1.1). Together with the results from immunostaining the micropylar guidance mutants and the knockdown analysis, we concluded that AtLURE1 peptides are the micropylar pollen tube attractants in *A. thaliana*.

### Various Mutations of *AtLURE1* Genes Have Occurred Independently in *A. thaliana* Accessions

We showed that *AtLURE1* (*CRP810_1*) genes encode pollen tube attractants for micropylar guidance. However, we found two loss-of-function mutations (*AtLURE1.5* and *1.6*) among the six *AtLURE1* genes in the analysis of the Col-0 accession. To examine whether such loss-of-function mutations of the *AtLURE1* genes are frequently observed in other accessions, we attempted to determine the nucleotide sequences of *AtLURE1.1* to *1.6* using PCR-based direct sequencing from 12 different accessions (ecotypes) of *A. thaliana*. We obtained 60 genomic sequences of the *AtLURE1* genes (Figure S6; Table S2). Each accession had at least four copies of *AtLURE1* genes although it was unknown whether a set of all paralogous *AtLURE1* genes was determined. We found many variations in nucleotide and amino acid sequences, some of which are likely to cause a loss of function, including a base substitution in a splicing acceptor site (Cvi-0 *AtLURE1.3*), the substitution of conserved cysteine residues (Kondara *AtLURE1.5*), and stop mutations (Ms-0 and Bur-0 *AtLURE1.5*). In contrast to the Col-0 accession, we also found putative functional *AtLURE1.5* and *AtLURE1.6* sequences which have no stop mutation and no mutation in conserved cysteines (Table S2). Furthermore, premature stop codons have been predicted in two accessions for *AtLURE1.3* and in eight accessions for *AtLURE1.5* according to sequence information of 80 accessions from the 1001 Genome Project [29]. These results suggest that a loss of function of some *AtLURE1* genes occurred independently in various accessions. *AtLURE1* genes without loss-of-function mutations must be sufficient to maintain each ecotype.

### Evolutionary Characterization of LURE1 Peptides from *A. thaliana* and *A. lyrata*

The *AtLURE1* genes diverged after the split of *A. thaliana* and *A. lyrata* and formed a species-specific gene cluster (Figure 2A and 2B). To provide further insight into the evolutionary and physiological properties of the AtLURE1 peptides and the AlCRP810_1 (AlLURE1) peptides in the orthologous clade, we performed the following two analyses: an evolutionary analysis to examine whether any significant positive selection was detected and a physiological pollen tube attraction assay to examine species specificity or preferentiality in pollen tube attraction.

The rates of nonsynonymous substitutions versus synonymous substitutions (dN/dS) were calculated for the *AtLURE1* and *AlLURE1* genes, as well as other duplicated *DEFL* genes (Tables S1 and S3), using approximate methods [30] available in the PAML package [31]. Although the dN/dS values of some of the duplicated *DEFL* genes were greater than 1, suggesting the occurrence of positive selection, we could detect no significant positive selection between the *AtLURE1* and *AlLURE1* genes. We also calculated the dN/dS values between a putative original copy *AtLURE1.1* and the other paralogous *AtLURE1* genes (*AtLURE1.2–1.5*) within *A. thaliana*. The values were not greater than 1 in any comparisons (Table S3). Next, we performed tests for positive selection on any of the individual codons and along any of the branches. A maximum likelihood tree of five *AtLURE1* and six *AlLURE1* genes that have no premature stop codon was used in these analyses. For the individual codons, there was no indication that the dN/dS values were significantly greater than 1 (the lowest probability of rejecting the null hypothesis of neutral evolution was >0.19) using HyPhy implemented in MEGA [32]. For the branches, the test was conducted using the program codeml available in the PAML package [31]. The program calculated the individual dN/dS values for each branch and a single average dN/dS value. By comparing the likelihood ratio for the two values with a chi-square test, the probability was not significant (*p*>0.41). This suggested that there was not likely to be variation in the dN/dS values and thus the possibility of positive selection along any of specific branches. Consequently, we did not detect evidence for positive selection in pairwise comparisons of overall sequences, on specific codons, and along specific branches of *AtLURE1* and *AlLURE1* genes.

Since no evidence that the *AtLURE1* and *AlLURE1* genes are evolving under positive selection was observed, we conducted the McDonald-Kreitman test [33] to test a hypothesis that *AtLURE1* genes have evolved under neutral evolution. According to the neutral theory, the dN/dS values between the two species should be equivalent to the rate of nonsynonymous to synonymous polymorphism within *A. thaliana*. Although one-to-one orthologous relationship between *AtLURE1* and *AlLURE1* genes could not be defined, we tried the test for coding sequences of each *AtLURE1* gene of the *A. thaliana* accessions (Table S2) and *AlLURE1.5* gene as a representative from *AlLURE1* genes. The test was not significant for *AtLURE1.1*, *1.2*, *1.3*, *1.4*, and *1.6* (*p*>0.69, 0.75, 1.0, 0.16, and 1.0, respectively; Table S4) except for *AtLURE1.5* (*p*<0.05; Table S4). We also observed similar results when compared to other *AlLURE1* genes as a representative (unpublished data). These results implied that most *AtLURE1* genes have evolved by drifting neutrally.

We found that divergence of amino acid sequence between AtLURE1 and AlLURE1 peptides was higher among the subgroups of multiply duplicated DEFL peptides analyzed (Table S1). Since we did not detect evidence for positive selection for the LURE1 peptides of *A. thaliana* and *A. lyrata*, it is suggested that the AtLURE1 peptides have undergone relatively rapid sequence change under neutral evolution, resulting in higher divergence of the sequences, and gene duplication after the divergence of the two species.

### AtLURE1 Has a Species-Preferential Pollen Tube Attraction Activity

**Figure 6:**
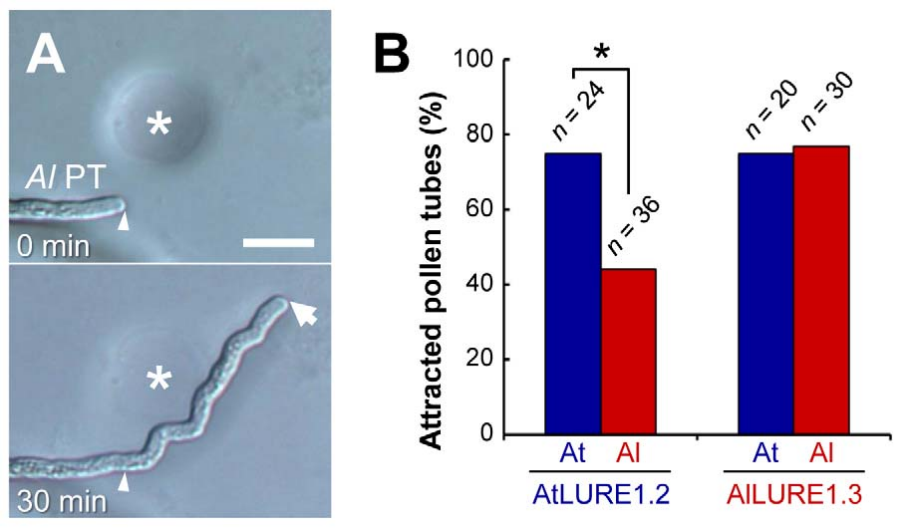
Pollen tube attraction of *A. lyrata* and species preferentiality of AtLURE1 and AlLURE1 peptides. (A) A representative example of an attracted pollen tube of *A. lyrata* (*Al* PT) to recombinant AlCRP810_1.3 (AlLURE1.3). Arrowheads mark the position of the pollen tube tip when a gelatin bead (asterisk) was placed. An arrow indicates the tip of the pollen tube. Scale bar, 20 µm. (B) Attraction activity of AtLURE1.2 (5 µM) and AlLURE1.3 (50 µM) to *A. thaliana* and *A. lyrata* pollen tubes. The data represent the frequencies of the total number of attracted pollen tubes (*n*) in at least three assays. An asterisk indicates a significant difference according to Fisher exact test (*p = 0.033). Note that the effective rate for each recombinant peptide is unknown because of differing efficiencies in peptide refolding.

Next, we investigated reciprocal pollen tube attraction activities of AtLURE1 and AlLURE1 peptides in *A. lyrata* and *A. thaliana*. We were able to prepare only one peptide from *A. lyrata*, being the predicted mature peptide of AlLURE1.3, as a recombinant peptide. We confirmed the ability of AlLURE1.3 to attract *A. lyrata* pollen tubes (Figure 6A), suggesting that AlLURE1 peptides are also pollen tube attractants in *A. lyrata*. The attraction frequencies of a representative recombinant peptide from *A. thaliana*, AtLURE1.2, were 75% (*n* = 24) for *A. thaliana* pollen tubes and 44% (*n* = 36) for *A. lyrata* pollen tubes, whereas those of AlLURE1.3 were 75% (*n* = 20) and 77% (*n* = 30), respectively, under the same conditions (Figure 6B). These results suggested that the AtLURE1 peptide had species-preferentiality, whereas the AlLURE1 peptide did not. Additionally, we showed that the AtLURE1.2 peptide did not show significant attraction of *T. fournieri* pollen tubes when examined at 40 nM (13%, *n* = 38) and 50 µM (14%, *n* = 21), indicating specific attraction activity of the peptide depending on evolutionary distance.

### Breakdown of Reproductive Isolation Barriers by Transforming the *AtLURE1* Gene into *Torenia*

**Figure 7:**
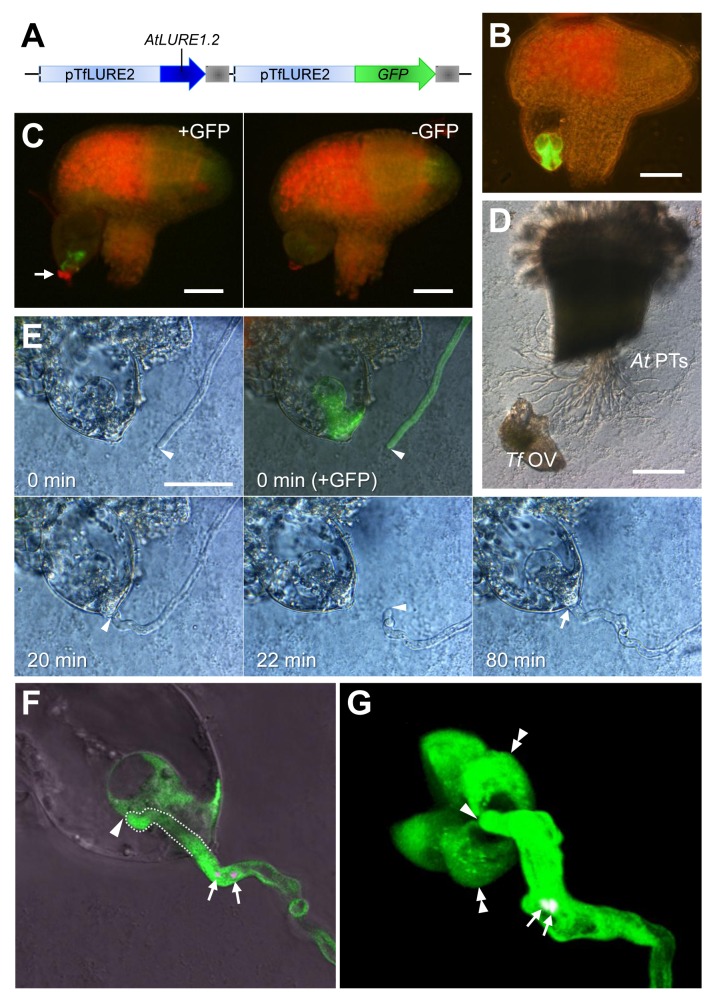
Interspecific pollen tube attraction assay using transgenic *T. fournieri* ovules and *A. thaliana* pollen tubes. (A) Schematic of the sequences introduced into *T. fournieri*. The *TfLURE2* promoter (pTfLURE2) was used to express both *AtLURE1.2* and *GFP* genes in synergid cells. The sequence of the *AtLURE1.2* gene is a genomic sequence from the translation initiation codon to the putative 3′ untranslated region. Gray boxes indicate *nopaline synthase* terminators. (B) GFP expression in two synergid cells of the transgenic *T. fournieri* ovule. Scale bar, 50 µm. (C) Immunostaining of transgenic *T. fournieri* ovules with anti-AtLURE1.2 (CRP810_1.2) antibodies. Red Alexa Fluor fluorescence for AtLURE1.2 (arrow) was observed at the micropylar surface of the embryo sac of the ovule labeled with GFP (left panel). In the ovule without GFP labeling (right panel), a weaker pseudo-signal was observed at the micropylar surface of the embryo sac. Scale bars, 50 µm. (D) An overview of the pollen tube attraction assay. Pollen tubes of *A. thaliana* (*At* PTs) are observed emerging from a cut style and growing across the medium. A manipulated *T. fournieri* ovule (*Tf* OV) was placed near the out-growing pollen tubes. Scale bar, 200 µm. (E) Pollen tube attraction toward the synergid cell of a transgenic *T. fournieri* ovule. Arrowheads indicate the position of the tip of the *A. thaliana* pollen tube. At 0 min, an ovule was placed in front of the tip of the pollen tube using a glass needle. The ovule expressed GFP in the synergid cell, indicating co-expression of the AtLURE1.2 peptide. Green fluorescence in the pollen tube indicates pollen-expressed *pLAT52::GFP*. The pollen tube was attracted to the ovule (20 min). After shifting the position of the transgenic ovule (22 min), the pollen tube redirected itself once again toward the micropyle (80 min). The arrow indicates the ovular micropyle penetrated by the attracted pollen tube. Scale bar, 50 µm. (F) A confocal laser scanning microscopic (CLSM) image of the transgenic *T. fournieri* ovule shown in (E), demonstrating that the tip of the *A. thaliana* pollen tube has fully penetrated the transgenic embryo sac (arrowhead). The pollen tube within the embryo sac is delineated by a dashed line. Note that discharge of sperm cells (arrows, sperm nuclei labeled with *pHTR10::HTR10:mRFP*) did not occur. (G) A projection of a 3-D reconstruction from CLSM images of cytosolic GFP-expressing synergid cells in the transgenic *T. fournieri* ovule and an attracted *A. thaliana* pollen tube (*pLAT52::GFP*×*pHTR10::HTR10:mRFP*). The arrowhead, double arrowheads, and arrow indicate the tip of the pollen tube, two synergid cells, and sperm nuclei, respectively. The projection is an oblique perspective viewed from the micropylar end of the embryo sac. The pollen tube entered the embryo sac at the filiform apparatus, which is surrounded by two synergid cells and an egg cell (left third part lacking GFP staining, adjacent to the two synergid cells).

If AtLURE1 peptides are in fact key molecules in the establishment of reproductive isolation, one may be able to break this barrier by introducing *AtLURE1* genes into other plant species. Thus, we produced transgenic *T. fournieri* plants expressing an AtLURE1 peptide and examined whether reproductive isolation can be overcome in this manner. Despite a large evolutionary distance between *A. thaliana* (Brassicaceae, Brassicales) and *T. fournieri* (Linderniaceae, Lamiales) [34], AtLURE1 peptides were partially similar to TfLUREs, including the alignment of conserved cysteines, the CXC motif at the C-terminus (Figure S7A), and specific expression in the synergid cell. The remaining amino acids, however, diverged considerably, demonstrating the loss of a conserved glycine residue for the gamma-core motif, which is generally observed in antimicrobial CRPs. Consistent with this divergence, TfLUREs did not attract pollen tubes of *A. thaliana* (Figure 5C and 5D). We then designed a transgenic plant co-expressing *AtLURE1.2* and cytosolic *GFP* specifically within the synergid cell of *T. fournieri*, under the control of the *TfLURE2* promoter (Figures 7A and S7B). In transgenic T_1_ heterozygous *T. fournieri* plants, we confirmed the specific expression of GFP in the synergid cell (Figure 7B) and the secretion of AtLURE1.2 peptide to the micropylar end of GFP-expressing synergid cells via immunostaining (Figure 7C). Using the transgenic *T. fournieri*, we performed an interspecific pollen tube attraction assay, in which manipulated ovules of the transgenic *T. fournieri* were placed close to the tip of *A. thaliana* pollen tubes in vitro (Figure 7D). The ovule was applied twice to one pollen tube to determine whether the ovule consistently attracted the pollen tube. Strikingly, the GFP-labeled transgenic *T. fournieri* ovules strongly attracted *A. thaliana* pollen tubes in both trials (100%, *n* = 11) (Figure 7E), whereas non-labeled ovules did not (0%, *n* = 11). The attraction was also precise, in that pollen tubes were able to redirect themselves toward the micropylar end of embryo sacs that had been shifted to a slightly different position. Strikingly, all of the attracted pollen tubes that continued to grow (*n* = 9) penetrated the embryo sac of *T. fournieri* (Figure 7F and 7G), through the region of the filiform apparatus (thickened cell wall) of the synergid cell, as reported in normal fertilization [35]. These results demonstrated that the heterologous expression of a single AtLURE1 attractant peptide alone is sufficient to overcome interspecific barriers in micropylar pollen tube guidance and penetration of the embryo sac, despite a large evolutionary distance between organisms.

## Discussion

### Mechanistic Insights into Micropylar Pollen Tube Guidance Mediated by LURE Peptides

Pollen tube growth and guidance have been intensively studied in *A. thaliana*, but the key attractant molecule has not been identified [36]. Here, we identified paralogous *DEFL* genes forming the sole species-specific *DEFL* gene cluster in *A. thaliana*, compared to its close relative *A. lyrata*, and found evidence that these *DEFL* genes, designated the *AtLURE1* genes, encode pollen tube attractants in *A. thaliana*. AtLURE1 peptides are small, secreted DEFL peptides that are specifically expressed in synergid cells (Figure 3). In vivo data showed that AtLURE1 peptides diffused to the surface of the funiculus of the ovule through the micropyle (Figure 3F) and that downregulation of these peptides impaired micropylar guidance (Figure 4). Moreover, recombinant AtLURE1 peptides expressed in *E. coli* attracted pollen tubes in a concentration-dependent and species-preferential manner in vitro (Figures 5 and 6B). These results provide concrete evidence that AtLURE1 peptides are pollen tube attractants in *A. thaliana* necessary for micropylar guidance.

Specific and abundant attractant peptide expression in the synergid cell is suggested in *A. thaliana* (Figure 3, [21]), as in *T. fournieri* [17]. *AtLURE1* gene duplication appears to have contributed to the increased amount of *AtLURE1* mRNA (Figure 3B). We propose that highly homologous AtLURE1 peptides (80%–95% amino acid sequence identity) are functionally redundant because each recombinant peptide showed attraction activity in vitro (Figure 5), we could find no combination effect among them (unpublished data), and heterologous expression of the AtLURE1.2 peptide in *T. fournieri* showed sufficient activity to attract *A. thaliana* pollen tubes into the embryo sac (Figure 7). Abundant expression might be a common characteristic of diffusible attractants derived from the synergid cell. The synergid cell is morphologically unique, showing characteristics typical of transfer cells, which are active in secretion and/or molecule uptake [37]. The synergid cell has been proposed to be a general source of pollen tube attractants in flowering plants due to its conserved morphology [37],[38]. As proposed after the laser-mediated ablation of synergid cells in *T. fournieri* [39], the amount of attraction signal may be correlated with the effective range of the signal. The endogenous localization of AtLURE1 peptides, as revealed by the immunostaining, suggests that the peptides diffused to the surface of the ovular funiculus (∼250 µm from the basal edge of the synergid cell). This is consistent with previous estimates of the effective range of pollen tube attraction in *A. thaliana*, based on the behavior of pollen tubes in vivo and in vitro [36],[40]. The abundant expression and active secretion of AtLURE1 peptides might be important for retaining a sharp peptide concentration gradient toward the synergid cell at a sufficient distance.

Like TfLUREs, AtLURE1 peptides are DEFL peptides, although their amino acid sequences have diverged considerably (Figures 2C and S7A). The LURE peptides of *Torenia* [17],[41] and *Arabidopsis* showed similar alignments of cysteines, which are typical of plant and insect defensins, including a CXC motif at the C-terminus [11]. A glycine in the gamma core of antimicrobial peptides is contained in *Torenia* LUREs [17],[41], but not in *Arabidopsis* LURE1 peptides. Because *Arabidopsis* (Brassicaceae) and *Torenia* (Linderniaceae) are quite divergent dicots, DEFL peptides might function as general pollen tube attractants in dicotyledonous plants. Although searching for attractants in other plant species is difficult due to low homologies, our study suggests that LURE-type DEFL peptides, which are abundantly and specifically expressed in the synergid cell, are candidate attractants. In the monocot *Z. mays*, another type of small secreted peptide, EGG APPARATUS 1 (EA1), was recently shown to be an attractant molecule derived predominantly from the synergid cell [42]–[44]. GFP-fused *Z. mays* EGG APPARATUS 1 (ZmEA1) appears to diffuse toward the micropylar opening as the ovule develops [42]. EA1-like genes comprise a large family in monocots [45]. Monocots may use non-DEFL peptides as attractants, although EA1-like genes are not likely to be expressed in synergid cells in rice [46].

Various CRPs, including DEFL peptides, are involved in the cell–cell communications underlying male–female interactions during plant reproduction [14]. The suggestion has been made that nearly 200 of 825 *CRP*s are expressed in the *A. thaliana* female gametophyte [21]. Not only AtLURE1 peptides but also other DEFL peptides and CRPs could be involved in cell–cell communication among gametophytic cells. Since the downregulation of AtLURE1 peptides partly inhibited micropylar guidance (Figure 4), additional attractant peptides may be derived from the synergid cell. As we observed abnormal pollen tube guidance in the micropylar guidance mutants at higher frequencies than in the RNAi lines, such additional attractants may be downregulated in these mutants. In fact, we found that some additional genes belonging to the CRP810 subgroup, which consists of 16 DEFL peptides in *A. thaliana* (Figure S8), were downregulated in the *myb98* mutant ovule [21]. Therefore, we performed qRT-PCR analysis for all of the *CRP810* genes and confirmed that some of these were expressed in the pistil at high levels as *AtLURE1* genes (Figure S9). Although whether they function as attractant peptides is unknown, our results suggest the possibility that many more pollen tube attractant peptides are expressed in the synergid cell, which may be required to efficiently attract pollen tubes to all female gametophytes. Nonetheless, our key finding will considerably accelerate the study of the directional control of tip-growing pollen tubes, the determination of precise spatiotemporal control of pollen tube attraction to the ovule, and identification of the receptor(s) of pollen tube attractants, which has not yet been identified [36].

### The Sole Species-Specific Cluster of *DEFL* Genes in *A. thaliana* Encodes Micropylar Pollen Tube Attractants

Using phylogenetic and synteny analyses, the *AtLURE1* (*CRP810_1*) and *AlCRP810_1* genes were suggested to form species-specific tandem arrays after the divergence of the two species (Figure 2A and 2B), possibly via gene duplication and/or gene conversion [47],[48]. Other clusters of paralogous *DEFL* genes in *A. thaliana* (≥four genes with ≥90% bootstrap values) were suggested to be formed before speciation occurred (Figures 1 and S1A), and syntenic conservation was observed between these *DEFL* genes from *A. thaliana* and *A. lyrata* (Figure S1B). Post-divergence, many genes have undergone lineage-specific duplication in *A. thaliana* [19]; however, among the 317 *DEFL* genes, the *AtLURE1* attractant genes represent the only recently formed, species-specific cluster. Moreover, some subgroups of mammalian β-defensin gene lineages exist specifically in certain species and are preferentially expressed in the male reproductive tract [49]. Clarifying whether these β-defensins play a role in fertility would be of interest.

Rapid gene turnover has been reported in multigene families involved in disease, including the effector genes of *Drosophila* (e.g., antimicrobial peptides [8]), the recognition genes of the sea urchin *Strongylocentrotus purpuratus* (e.g., Toll-like receptors [7]), and the effector genes for avirulence activity of the rice blast fungal pathogen *Magnaporthe oryzae* [9], possibly due to a coevolutionary arms race. Our work demonstrates that genes involved in direct male–female interactions in sexual reproduction can also increase rapidly in copy number during speciation. To our knowledge, such a species-specific gene cluster directly involved in male–female interactions has not been reported. The relationship between multicopy factors for sexual reproduction and their rapid molecular evolution is an important issue to be addressed. In addition to the indicated lineage-specific duplication, AtLURE1 peptides showed lower homology (∼70%) among multiply duplicated DEFL peptides when compared to orthologous *A. lyrata* DEFL peptides. This suggests the rapid evolution of *AtLURE1* genes possibly by high mutation rate under neutral genetic drift because we were unable to detect any evidence of positive selection. Similarly no evidence for positive selection has been found in multicopy antimicrobial peptides in *Drosophila*, but they might not require functional change between species due to a lack of coevolutionary arms race [6],[8],[50]. This seems to be different from the case of *AtLURE1* genes, which encode peptides showing species-preferential activity.

Various mutations, including cysteine substitutions and stop mutations, were observed in different ecotypes of *A. thaliana* (Figure S6; Table S2). A cysteine substitution in AtLURE1.5 found in the Col-0 line was shown to impair attraction of the pollen tube (Figure 5C and 5D). *AtLURE1* gene duplication is likely to confer a relaxation of functional constraints due to gene redundancy [51]. As long as some population of attractant peptides interacts with a receptor, functional changes such as specificity for a receptor of the other population of peptides could be permitted with normal pollen tube guidance. One may speculate that multiple attractant genes may be important for reinforcing species specificity by sequence divergence without loss of correct ligand–receptor pairing.

### Non-Cell Autonomous Regulation of Pollen Tube Attraction

In addition to the mutant ovules of *myb98*, those of *ccg* and *maa3* could not secrete AtLURE1 peptides, as shown by immunostaining (Figure 3G and 3H), suggesting that attractant secretion occurs via CENTRAL CELL GUIDANCE (CCG)- and MAA3-related regulatory pathways. CCG is a transcriptional regulator in the central cell (but not the synergid cell) that functions in micropylar guidance. Regarding the phenotype of the *ccg* mutant, whether the central cell directly attracts pollen tubes by emitting a diffusible signal or indirectly controls pollen tube guidance via cell–cell communication between the central cell and synergid cell has been debated [52]. Our results are consistent with the latter model, in which CCG in the central cell indirectly controls the expression of AtLURE1 peptides in the synergid cell. Gametic cells (the egg cell and central cell) communicate with each other, and likely control cell specificity in the synergid and antipodal cells during female gametophyte development [53]–[56]. Our results suggest that CCG in the central cell is required for full functioning of the synergid cell. The identification of additional downstream genes regulated by CCG and upstream genes regulating attractant production will clarify how the central cell controls attractants secreted from the synergid cell at the molecular level.

MAA3 helicase is essential for normal development of the female gametophyte and is likely generally involved in RNA metabolism in various cell types [26]. Although the phenotype of the *maa3* ovule includes smaller nucleoli in two polar nuclei and failure of fusion of the polar nuclei in the central cell [57], whether the synergid cell and/or the central cell are responsible for abnormal pollen tube guidance in *maa3* remains unclear. AtLURE1 peptides were not secreted from the *maa3* ovule, as shown by immunostaining (Figure 3G and 3H). This finding implies that attractant production could be regulated by MAA3-mediated RNA metabolism. RNA metabolism, including rRNA biogenesis, is important for female gametophyte development [58]–[60]. Complex regulation via RNA metabolism and synergid cell–central cell interactions may render female gametophytic cells fully mature, after which the expression of attractant peptides, including AtLURE1 peptide, is initiated.

### AtLURE1 Peptides May Overcome Pre-zygotic Reproductive Barriers

Interspecific cross-pollination with *A. thaliana* pistils was shown to cause mis-targeting of some pollen tubes at the micropylar guidance step [61]. Consistent with this idea, AtLURE1.2 peptide showed species-preferentiality in its attraction activity when pollen tubes from *A. thaliana* and *A. lyrata* were examined (Figure 6). Unexpectedly, AlLURE1.3 peptide showing low sequence identity (∼70%) with AtLURE1 peptides attracted pollen tubes from both *A. thaliana* and *A. lyrata* in a similar frequency (Figure 6). This might be related to evolutionary pattern of receptor(s) for the LURE peptides between the two species. Elucidating coevolution of the LURE peptides and the receptors of both species would shed light on mechanism of formation of reproductive barriers in pollen tube guidance. In *T. fournieri* and its close relative *T. concolor*, rapid molecular evolution of *TfLURE1* and *TcLURE1* was likely directly involved in the species preferentiality of pollen tube attraction [41],[62]. The expression of LURE attractant peptides at the last step of pollen tube guidance is likely to be one of the more severe reproductive isolation barriers. This was supported by the fact that a single *AtLURE1* gene introduced into *T. fournieri* was sufficient to attract competent *A. thaliana* pollen tubes to the *T. fournieri* embryo sac. The degree of tracking precision shown by these pollen tubes was greater than expected: pollen tubes of *A. thaliana* followed the filiform apparatus (only 10 µm in surface diameter) of manipulated ovules of transgenic *T. fournieri*, as is normally observed when *T. fournieri* pollen tubes approach the embryo sac [38]. Recently, synthetic ZmEA1 peptide was shown to attract maize pollen tubes, and heterologous expression of ZmEA1-GFP in the synergid cell of *A. thaliana* resulted in the attraction of maize pollen tubes toward the micropyle [44]. These results may open the way for new technologies to overcome reproductive barriers in flowering plants, although further research is required to identify other key factors preventing heterologous fertilization. Our research into the AtLURE1 peptide further showed that heterogeneous pollen tubes can enter the embryo sac normally through the region of the filiform apparatus. This suggests that pollen tube entrance into the embryo sac does not involve any other severe species-recognition mechanism, at least when combining the *T. fournieri* embryo sac with an *A. thaliana* pollen tube. The succeeding steps in fertilization, being pollen tube recognition and triggered rupture by the synergid cell, may show severe species-specificity [18],[63]. *FERONIA* [63] and *ZmES4* [18] expressed in the synergid cell are good candidates for mediating species recognition during these steps.

Some post-zygotic isolation barriers [64] involved in hybrid dysfunction have been identified in plants through genetic studies [65]–[67]. Rapidly changing reproductive genes, including *AtLURE1*, *TfLURE1*, *TcLURE1* [41],[62], ZmEA1 [42],[45], *FERONIA* [63], and *ZmES4* [18], represent potential pre-zygotic isolation barriers during the final steps of pollen–pistil interactions. In pollen–pistil interactions, the final steps show much greater species preferentiality [38]. Identifying their partner molecules, including the receptor of LURE, is important to understanding the mechanism of speciation and plant reproduction.

## Materials and Methods

Additional details are provided in Text S1. The sequences of the primers used in this study are given in Table S5.

### Plant Materials

For all experiments except the sequence analysis, accession Col-0 was used as wild-type *A. thaliana*. For the sequence analysis of 12 *A. thaliana* accessions, Cvi-0 (CS22614), Est-1 (CS22629), Mr-0 (CS22640), Tsu-1 (CS22641), Nok-3 (CS22643), Fei-0 (CS22645), Ts-1 (CS22647), Pro-0 (CS22649), Kondara (CS22651), Ms-0 (CS22655), Bur-0 (CS22656), and Ws-2 (CS22659), seeds were obtained from Sumie Ishiguro (Nagoya University). The *myb98* (SALK_020263) and *ccg* (SALK_077907) mutants are T-DNA insertion lines [68]. The T-DNA insertion mutant *maa3* was provided by Kentaro K. Shimizu (University of Zurich). *A. lyrata* (accession CS22696) was provided by Akira Kawabe (Kyoto Sangyo University).

### Plant Transformation

T-DNA constructs were introduced into *Agrobacterium tumefaciens* strain GV3101 and then transformed into plants using the floral dip method for *A. thaliana* [69] or the leaf disc method for *T. fournieri* [70], with some modifications. Transformed seeds or leaf discs were selected on medium containing antibiotics.

### Sequence Determination

*A. thaliana* (Col-0) nucleotide sequences were obtained from TAIR (http://www.arabidopsis.org/) and confirmed by sequencing. Predicted signal peptide cleavage sites in CRP810_1.1 to _1.5 (AtLURE1.1 to 1.5) were determined from their full-length amino acid sequences using SignalP (http://www.cbs.dtu.dk/services/SignalP/). Orthologous *DEFL* genes in *A. lyrata* were identified by BLAST search at the Department of Energy Joint Genome Institute (DOE JGI) (http://genome.jgi-psf.org/Araly1/Araly1.home.html).

Synteny analysis between the genomic sequences of *A. thaliana* and *A. lyrata* containing *CRP810_1* genes was performed using genomic data from the two species. Two gaps on scaffold_8 (4222301–4251300) from *A. lyrata* showing synteny with the region of *CRP810_1.2* to *1.6* were sequenced to confirm that no *Al CRP810_1* gene existed in this scaffold (contig). The genes *CRP810_1.8*, *1.9*, and *1.10* were on a small scaffold (scaffold_97 and scaffold_1021) that showed no synteny with the *A. thaliana* genome.

### Phylogenetic Tree Analysis

Phylogenetic analyses were done as described previously [62]. Full-length amino acid or genomic sequences (a start codon to a stop codon in the genomic DNA) were aligned using ClustalX [71]. Phylogenetic trees of the aligned sequences were drawn using the neighbor-joining (NJ) method, including bootstrapping [72] based on 1,000 replicates using ClustalX or MEGA 5 [32].

### Quantitative Real-Time RT-PCR

Total RNAs were purified from each tissue using RNAqueous-Micro (Ambion) with Plant RNA Isolation Aid (Ambion). Each tissue was homogenized in lysis solution. Following total RNA purification, reverse transcription reactions were carried out using SuperScript III Reverse Transcriptase (Invitrogen). Quantitative real-time PCR was performed using the cDNA and Power SYBR Green PCR Master Mix (Applied Biosystems) on an Applied Biosystems StepOnePlus Real-Time PCR System. To quantify the absolute expression levels of the *CRP810_1* genes, the standard curve method with template vectors of known copy number was used. To quantify the relative expression levels, the comparative C_T_ (ΔΔC_T_) method was used.

### Promoter GFP Analysis

Sequences 1,129, 665, 3,304, 1,789, and 1,957 bp upstream of the transcription start site were used as promoter sequences of *CRP810_1.1*, _*1.2*, _*1.3*, _*1.4*, and _*1.5*, respectively. To observe GFP fluorescence in mature ovules, flowers of the transformed plants were emasculated before anthesis. For epifluorescence microscopy, an inverted microscope (IX71, Olympus) with a 3CCD camera (C7780-20, Hamamatsu Photonics) was used. For confocal laser scanning microscopy, an IX71 equipped with a disk-scan confocal system (CSU10, Yokogawa) and EM-CCD camera (Cascade II:512, Roper) was used.

### Immunostaining

Recombinant CRP810_1.2 (AtLURE1.2) purified as described in the next section was prepared and used to immunize a rabbit. Purified pre-immune or anti-CRP810_1.2 antibodies (1∶1,000 dilution) and Alexa Fluor 488-conjugated anti-rabbit goat IgG (1∶1,000 dilution; Invitrogen) were used. For immunostaining, the carpel walls were removed from the pistil, resulting in ovules on the septum. The ovules were then fixed using 4% paraformaldehyde in PBS for 40 min or a 9∶1 mixture of ethanol and acetic acid overnight. The subsequent protocol was identical to that in a previous report [17]. For confocal imaging, an LSM 780 NLO (Zeiss) system was used.

### Purification of Recombinant Proteins and In Vitro Attraction Assay

The coding sequences of the predicted mature peptides of AtCRP810_1.1 to _1.5 (AtLURE1.1 to 1.5) and AlCRP810_1.3 (AlLURE1.3) were amplified from the cDNA of the pistil or genomic DNA and were cloned into pET-28a(+) (Novagen) using the *Bam*HI site to fuse a His tag to the N-terminus. Note that although the full sequence of AlLURE1.3 contains a premature stop codon in the signal peptide, the mature AlLURE1.3 peptide shows just two amino acid differences compared to its closest paralog, AlLURE1.9, and was capable of attracting *A. lyrata* pollen tubes. These expression vectors were transformed into *E. coli* strain BL21-CodonPlus (Stratagene). The His-tagged peptides were expressed at 37°C for 5 h and purified by metal affinity chromatography with HisTrap FF (for CRP810_1.2, _1.3, and _1.4; GE Healthcare) or TALON Metal Affinity Resins (for CRP810_1.1, _1.5, and CRP810_1.5-Y84C; Clontech). TfLURE1 and -2 were prepared as described previously [17]. The subsequent procedures, namely, refolding of the peptides, preparation of the gelatin beads, and criteria for judgment of the attracted pollen tubes, generally followed the procedure described previously for TfLUREs [17]. For the in vitro attraction assay, growth medium was used for the pollen tubes [27],[28].

### Generation of RNAi Constructs

The inverted repeat sequence from the *CRP810_1.2* (*AtLURE1.2*) coding sequence with the linker sequence was connected to the downstream region of the *MYB98* promoter and introduced into pENTR/D-TOPO (Invitrogen). This construct was transferred to the binary vector pGWB1 [73] using the LR recombination reaction (Invitrogen).

### Analysis of Pollen Tube Guidance in the Pistil and on Medium

For the analysis of pollen tube guidance in the pistil, carpel walls were removed from the pistil ∼1 d after flowering, and the resulting ovules and septum were stained with aniline blue without fixation. To evaluate abnormal pollen tube guidance around the micropylar opening, the number of ovules was counted only when the pollen tube(s) was observable from the funiculus to the ovule in the prepared slide.

For competition analysis of pollen tube guidance on medium, two ovules (e.g., a wild-type ovule and an RNAi ovule) were arranged with their micropylar openings close together under a stereomicroscope. After incubation at 22°C in the dark, the pollen tube first reaching the space between the two ovules was carefully observed. Next, the pollen tube was watched until either ovule attracted it to the micropyle. If neither ovule attracted it, that trial was designated as not determined (ND).

### Pollen Tube Attraction by *T. fournieri* Ovules Expressing *AtLURE1.2*

To generate the construct for *T. fournieri* expressing *AtLURE1.2* (Figure 7A), the *AtLURE1.2* genomic sequence (including the 3′ untranslated region) and the *GFP* sequence were connected to the downstream region of the *TfLURE2* promoter (Figure S7B), as identified by thermal asymmetric interlaced (TAIL)-PCR. The two sequences were ligated and then introduced into the binary vector pPZP211 [74]. The construct was transformed into selfed generations of *T. fournieri* cv. “blue and white.” Transformed T_1_ heterozygous plants were used for the interspecific pollen tube attraction assay. In the assay, *A. thaliana* pollen tubes grown through a cut style were incubated for 4–5 h on growth medium for *T. fournieri* [17], and then ovules were dissected from the transformed *T. fournieri* and manipulated using a glass needle under an inverted microscope (IX71, Olympus). For confocal imaging, an LSM 780 NLO (Zeiss) system was used.

### Accession Numbers

The accession numbers of the *CRP810_1* genes are as follows: *CRP810_1.1* (At5g43285), *_1.2* (At5g43510), *_1.3* (At5g43513), *_1.4* (At5g43518), *_1.5* (At5g43525), and *_1.6* (At5g43516, a pseudogene).

## Supporting Information

Figure S1 **Phylogenetic tree analysis and synteny analysis of** ***DEFL*** **gene clusters in** ***A. thaliana*** **and** ***A. lyrata*** **, related to** **Figure 2A and 2B** **.** (A) Phylogenetic trees of paralogous genes clustered in the *A. thaliana* genome and their orthologous genes in *A. lyrata* based on the coding region of their genomic sequences. The trees include three of 13 paralogous gene clusters shown in Figure 1 and Table S1 as representative clusters that contain tandemly arrayed genes in the genome, *CRP700* genes (*A. thaliana* trypsin inhibitor, *ATTI*), *CRP580* genes (low molecular weight, cysteine-rich, LCR), *CRP860* genes (*SCR*-like, *SCRL*), and their orthologous genes in *A. lyrata*. Only bootstrap values ≥90 are indicated. The scale shows the number of substitutions per site. *A. thaliana* genes are shown in blue while *A. lyrata* genes are shown in red. The underlined genes are tandemly arrayed genes in the genome and syntenic genes shown in (B). (B) Synteny analysis of the tandemly arrayed paralogous genes. Syntenic regions of the *ATTI*, *LCR*, and *SCRL* genes are shown. Blue, red, and gray arrows represent the loci of the *A. thaliana* (Col-0) genes, their syntenic genes in *A. lyrata*, and unrelated genes, respectively.
(TIF)

Figure S2 **Multiple alignments of CRP810_1 peptides of** ***A. thaliana*** **and** ***A. lyrata*** **, related to** **Figure 2C** **.** Multiple alignments of the full-length amino acid sequences of CRP810_1 peptides and their orthologs in *A. lyrata* (AlCRP810_1) and an assumptive sequence of mature peptide of AlCRP810_1.3 following the putative cleavage site. Black and gray backgrounds indicate amino acids conserved among nine and five or more sequences, respectively. The arrow indicates the position of the predicted cleavage sites. Asterisks mark conserved cysteine residues.
(TIF)

Figure S3 **Analysis of the specificity of the anti-CRP810_1.2 antibodies and negative controls by immunostaining, related to** **Figure 3F** **.** (A) Fluorescence microscopic images of immunostained ovules using anti-CRP810_1.2 antibodies and pre-immune IgG as a negative control. In the immunostained ovule fixed in a 9∶1 mixture of ethanol∶acetic acid (AcOH–EtOH), Green Alexa Fluor fluorescence was observed in a similar way to the ovule fixed in paraformaldehyde (Figure 3F). Pre-immune IgG was not bound to the micropylar opening of the ovule when the ovule was fixed in AcOH–EtOH or paraformaldehyde. Scale bar, 50 µm. (B) Immuno-dot blot analysis confirming the recognition capability of the anti-CRP810_1.2 antibody. 10 µl of purified recombinant His-tagged peptide was blotted onto a PVDF membrane (Immobilon-P, Millipore); the total amount of blotted peptide is shown on the left. The PVDF membrane was then treated with anti-CRP810_1.2 antibodies (primary antibody, 1∶10,000 dilution) and peroxidase-conjugated anti-rabbit goat IgG (secondary antibody, 1∶20,000 dilution; KPL). Signals detected using a chemiluminescent reagent (Immobilon Western Chemiluminescent HRP Substrate, Millipore; Light-Capture, ATTO) are represented as black dots. All His-tagged CRP810_1 peptides were detected in a concentration-dependent manner, while His-tagged TfLURE1 and 2 as controls were barely detected. Among the CRP810_1 peptides, the signals for CRP810_1.1, which showed lower identity to CRP810_1.2, and CRP810_1.5, which lacked a single conserved cysteine, are comparatively weak.
(TIF)

Figure S4 **Knockdown analysis of** ***CRP810_1*** **genes by RNAi, related to** **Figure 4** **.** (A) Schematic of the RNAi constructs. The upper graphic represents the T-DNA region between the right (RB) and left (LB) borders. The genes encoding neomycin phosphotransferase (*NPT*) and hygromycin phosphotransferase (*HPT*) confer resistance to kanamycin and hygromycin, respectively. The middle and lower graphics represent the structures of *CRP810_1*-RNAi (RNAi) and the linker (vector control). The *MYB98* promoter (pMYB98) was used to drive the RNAi sequence in the synergid cells. The linker sequence is nucleotides 72–1,067 of the *GUS* coding sequence. *CRP810_1.2*-antisense and -sense sequences can form dsRNA when the sequence is transcribed. pNos, *nopaline synthase* promoter; NosT, *nopaline synthase* terminator; p35S, cauliflower mosaic virus (CaMV) *35S* promoter. (B) Real-time qRT-PCR analysis of *CRP810_1*, *MYB98*, and *At4g08869/At4g08875* in the pistils of the RNAi and vector control lines. To confirm the specific downregulation of the *CRP810_1* genes, synergid-specific *MYB98* and the closest *CRP810* genes (*At4g08869* and *At4g08875*) were used. The relative quantities are the expression levels relative to that in the wild type. Two independent vector control lines and three independent RNAi lines were thought to be homozygous T_3_ lines because the siblings of each T_3_ plant were drug-resistant. Each expression level was normalized to that of *ACT2*. The data are the means and standard errors of three sibling plants. Asterisks indicate significant differences compared with the wild type according to Dunnett's test (*p*<0.05) by ANOVA.
(TIF)

Figure S5 **In vitro pollen tube attraction assay of** ***Arabidopsis*** **, related to** **Figures 5** **and** **6** **.** (A) In vitro pollen tube growth. Pollen tubes through the cut style were grown on medium using an in vitro system. Ovules were co-cultured because unknown protein(s) derived from the ovules could promote pollen tube growth and/or attraction. Around 5 h after the start of incubation, the pollen tube attraction assays were started. The picture shows pollen tubes emerging from the cut style and ovules 6 h after the start of incubation. Scale bar, 100 µm. (B) A gelatin bead manipulated with a glass needle. The gelatin bead was attached to the tip of the glass needle and placed on the medium through micromanipulation. Scale bar, 20 µm. (C) A gelatin bead placed in front of the tip of a pollen tube. The gelatin bead was slowly dissolved in the medium to spread the proteins embedded in it. Scale bar, 20 µm.
(TIF)

Figure S6 **Sequence variation of** ***AtLURE1*** **(** ***CRP810_1*** **) genes in various accessions of** ***A. thaliana*** **.** A phylogenetic tree of six *AtLURE1* (*AtLURE1.1* to *1.6*) from the Col-0 accession and 60 sequences from 12 other *A. thaliana* accessions based on the coding region of their genomic sequences. Each background color indicates subtrees containing *AtLURE1.1* to *1.6*. The scale shows the number of substitutions per site. The genes with asterisks are probably nonfunctional. Also see Table S2.
(TIF)

Figure S7 **Sequence comparison of TfLUREs and CRP810_1.2 peptides and the promoter sequence of** ***TfLURE2*** **.** (A) Putative mature peptides of TfCRP1 (TfLURE1) and TfCRP3 (TfLURE2), pollen tube attractants of *T. fournieri*, and CRP810_1.2 (AtLURE1.2) as a representative CRP810_1 peptide are shown. Asterisks mark conserved cysteine residues. (B) Upstream sequence of a translation start site of *TfLURE2*. A green highlight and yellow highlights indicate *cis*-element GTAACNT, which is suggested to be MYB98-binding sequence, and *cis*-element AACGT, which is necessary and sufficient for *AtLURE1.2* (DD2) expression in the synergid cell of *A. thaliana*, respectively [23]. Red letters mark 5′ untranslated region of *TfLURE2*.
(TIF)

Figure S8 **A multiple alignment of CRP810 peptides.** A multiple alignment of the full-length amino acid sequences of 16 CRP810 peptides in *A. thaliana*. Black and gray backgrounds indicate amino acids conserved among 16 and ≥eight sequences, respectively. Asterisks mark conserved cysteine residues. The accession numbers of the *CRP810* genes are as follows: *CRP810_2.1* (*At5g48515*), *_2.2* (*At5g48595*), *_2.3* (*At5g48605*), *_3.1* (*At4g08869*), _*3.2* (*At4g08875*), *_4* (*At5g50423*), *_5* (*At5g18403*), *_6* (*At5g18407*), *_7* (*At5g60805*), and *_8* (*At4g08485*).
(TIF)

Figure S9 **Expression analysis of** ***CRP810*** **genes in** ***A. thaliana*** **.** (A) A phylogenetic relationship (left) and absolute gene expression levels in the pistil (right) of the *CRP810* genes. The tree is a portion of a phylogenetic tree of 317 DEFL peptides. Asterisks mark genes downregulated in *myb98* according to a genome-wide analysis [21]. The bootstrap values more than 50 for the neighbor-joining (NJ) method are indicated as percentages. The scale shows the number of amino acid substitutions per site. Absolute quantity represents the copy number of cDNA per that of *MYB98* cDNA. The means and standard deviations of three independent experiments are shown. (B) Absolute gene expression levels for paralogous gene groups. For *AtLURE1* (*CRP810_1*), *CRP810_2*, and *_3*, the values are the sum of the absolute quantities for each paralogous gene. The *CRP810* genes were numbered from *CRP810_1* (*AtLURE1*) to *_8* according to the level of expression in each group.
(TIF)

Table S1 **Sequences of paralogous** ***DEFL*** **genes in** ***A. thaliana*** **and their orthologs in** ***A. lyrata*** **.**
(XLS)

Table S2 **Sequences of** ***AtLURE1*** **genes in various accessions of** ***A. thaliana*** **.**
(XLS)

Table S3 **Pairwise comparisons of** ***AtLURE1*** **and** ***AlLURE1*** **genes.**
(XLS)

Table S4 **McDonald-Kreitman tables for** ***AtLURE1*** **genes.**
(XLS)

Table S5 **Primer sequences.**
(XLS)

Text S1 **Supporting methods and references.**
(DOC)

## Abbreviations

CCG: CENTRAL CELL GUIDANCE
CRP: cysteine-rich peptide
DEFL: defensin-like
GFP: green fluorescent protein
MAA3: MAGATAMA3
qRT-PCR: quantitative reverse transcription PCR
ZmEA1: *Zea mays* EGG APPARATUS 1
ZmES4: *Zea mays* EMBRYO SAC 4
