# Supplementary figures and images for: A Species-Specific Cluster of Defensin-Like Genes Encodes Diffusible Pollen Tube Attractants in Arabidopsis
Source: PLoS Biol. 2012 Dec 18;10(12):e1001449. doi: 10.1371/journal.pbio.1001449 (PMC3525529; doi:10.1371/journal.pbio.1001449)

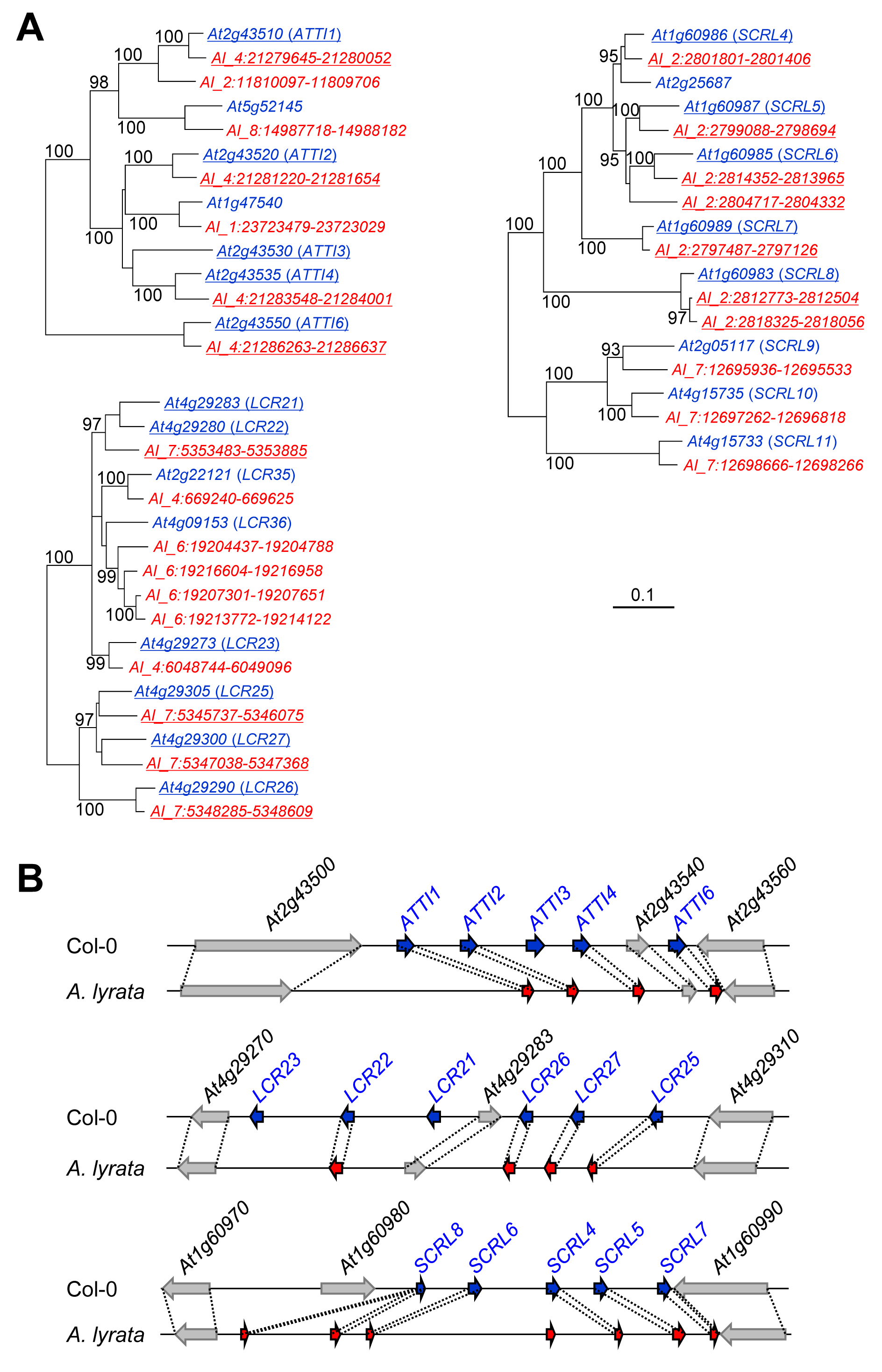

Supplement: Figure S1 — Phylogenetic tree analysis and synteny analysis of DEFL gene clusters in A. thaliana and A. lyrata , related to Figure 2A and 2B . (A) Phylogenetic trees of paralogous genes clustered in the A. thaliana genome and their orthologous genes in A. lyrata based on the coding region of their genomic sequences. The trees include three of 13 paralogous gene clusters shown in Figure 1 and Table S1 as representative clusters that contain tandemly arrayed genes in the genome, CRP700 genes (A. thaliana trypsin inhibitor, ATTI), CRP580 genes (low molecular weight, cysteine-rich, LCR), CRP860 genes (SCR-like, SCRL), and their orthologous genes in A. lyrata. Only bootstrap values ≥90 are indicated. The scale shows the number of substitutions per site. A. thaliana genes are shown in blue while A. lyrata genes are shown in red. The underlined genes are tandemly arrayed genes in the genome and syntenic genes shown in (B). (B) Synteny analysis of the tandemly arrayed paralogous genes. Syntenic regions of the ATTI, LCR, and SCRL genes are shown. Blue, red, and gray arrows represent the loci of the A. thaliana (Col-0) genes, their syntenic genes in A. lyrata, and unrelated genes, respectively. (TIF) [file pbio.1001449.s001.tif]

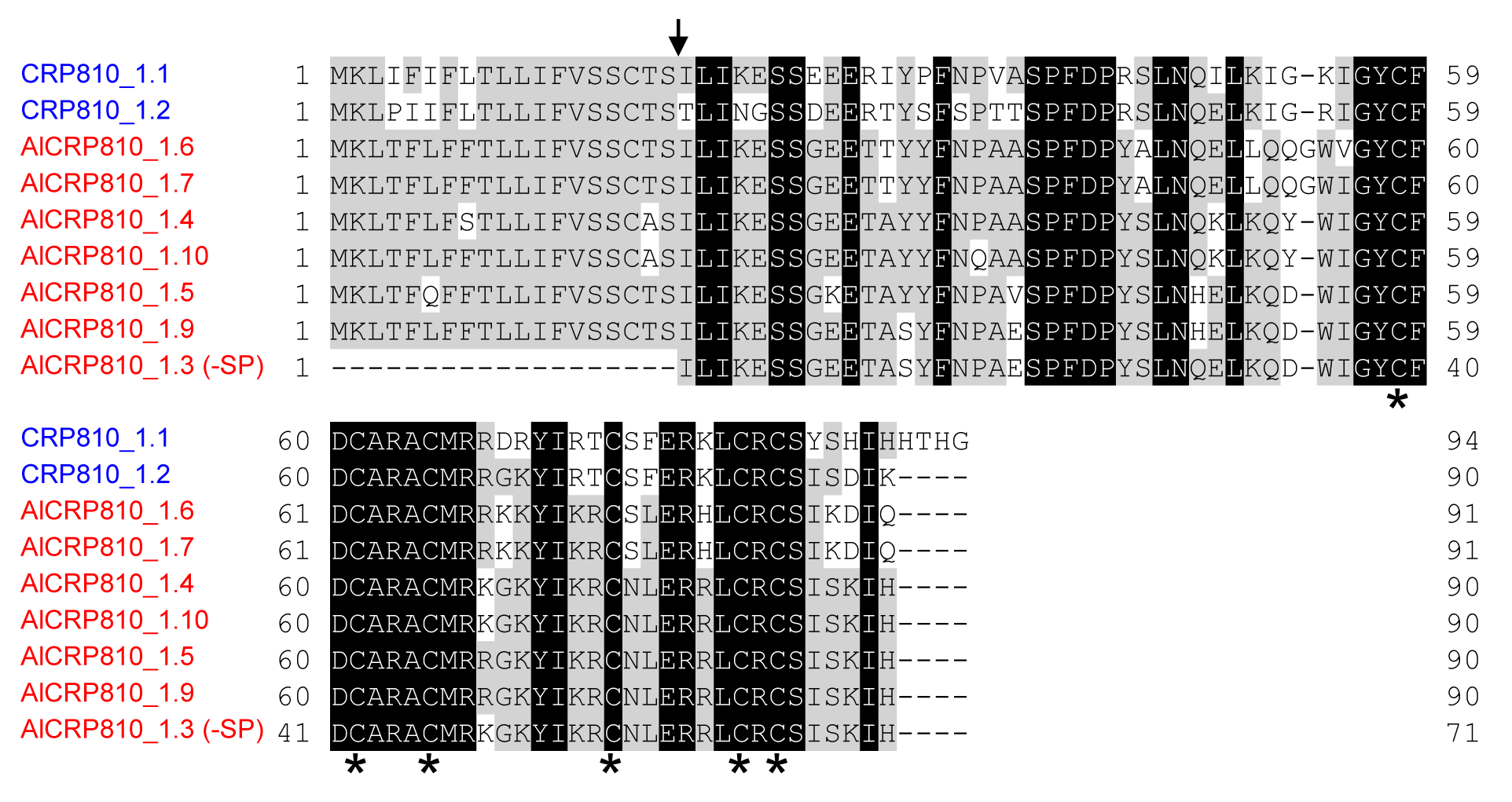

Supplement: Figure S2 — Multiple alignments of CRP810_1 peptides of A. thaliana and A. lyrata , related to Figure 2C . Multiple alignments of the full-length amino acid sequences of CRP810_1 peptides and their orthologs in A. lyrata (AlCRP810_1) and an assumptive sequence of mature peptide of AlCRP810_1.3 following the putative cleavage site. Black and gray backgrounds indicate amino acids conserved among nine and five or more sequences, respectively. The arrow indicates the position of the predicted cleavage sites. Asterisks mark conserved cysteine residues. (TIF) [file pbio.1001449.s002.tif]

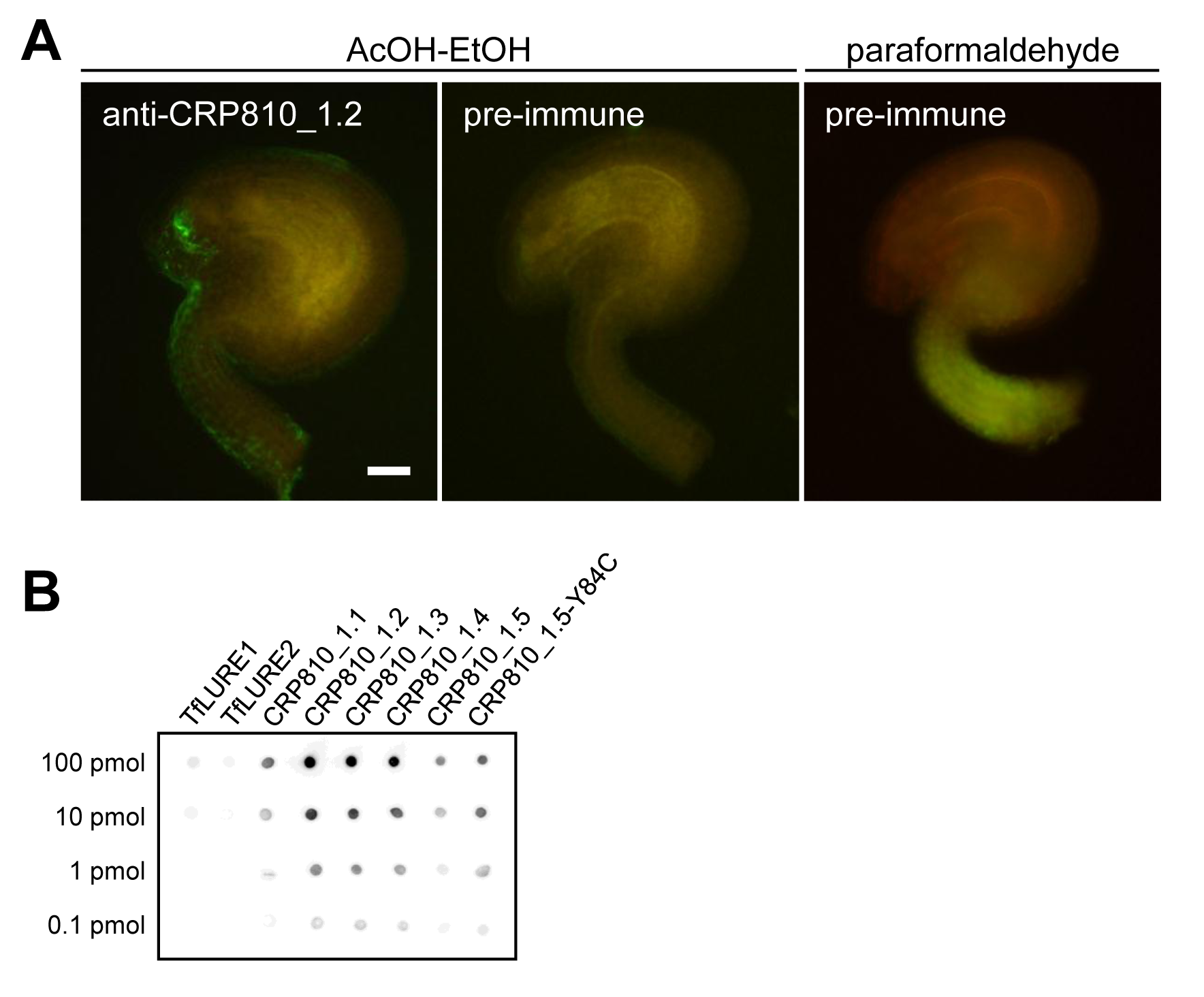

Supplement: Figure S3 — Analysis of the specificity of the anti-CRP810_1.2 antibodies and negative controls by immunostaining, related to Figure 3F . (A) Fluorescence microscopic images of immunostained ovules using anti-CRP810_1.2 antibodies and pre-immune IgG as a negative control. In the immunostained ovule fixed in a 9∶1 mixture of ethanol∶acetic acid (AcOH–EtOH), Green Alexa Fluor fluorescence was observed in a similar way to the ovule fixed in paraformaldehyde (Figure 3F). Pre-immune IgG was not bound to the micropylar opening of the ovule when the ovule was fixed in AcOH–EtOH or paraformaldehyde. Scale bar, 50 µm. (B) Immuno-dot blot analysis confirming the recognition capability of the anti-CRP810_1.2 antibody. 10 µl of purified recombinant His-tagged peptide was blotted onto a PVDF membrane (Immobilon-P, Millipore); the total amount of blotted peptide is shown on the left. The PVDF membrane was then treated with anti-CRP810_1.2 antibodies (primary antibody, 1∶10,000 dilution) and peroxidase-conjugated anti-rabbit goat IgG (secondary antibody, 1∶20,000 dilution; KPL). Signals detected using a chemiluminescent reagent (Immobilon Western Chemiluminescent HRP Substrate, Millipore; Light-Capture, ATTO) are represented as black dots. All His-tagged CRP810_1 peptides were detected in a concentration-dependent manner, while His-tagged TfLURE1 and 2 as controls were barely detected. Among the CRP810_1 peptides, the signals for CRP810_1.1, which showed lower identity to CRP810_1.2, and CRP810_1.5, which lacked a single conserved cysteine, are comparatively weak. (TIF) [file pbio.1001449.s003.tif]

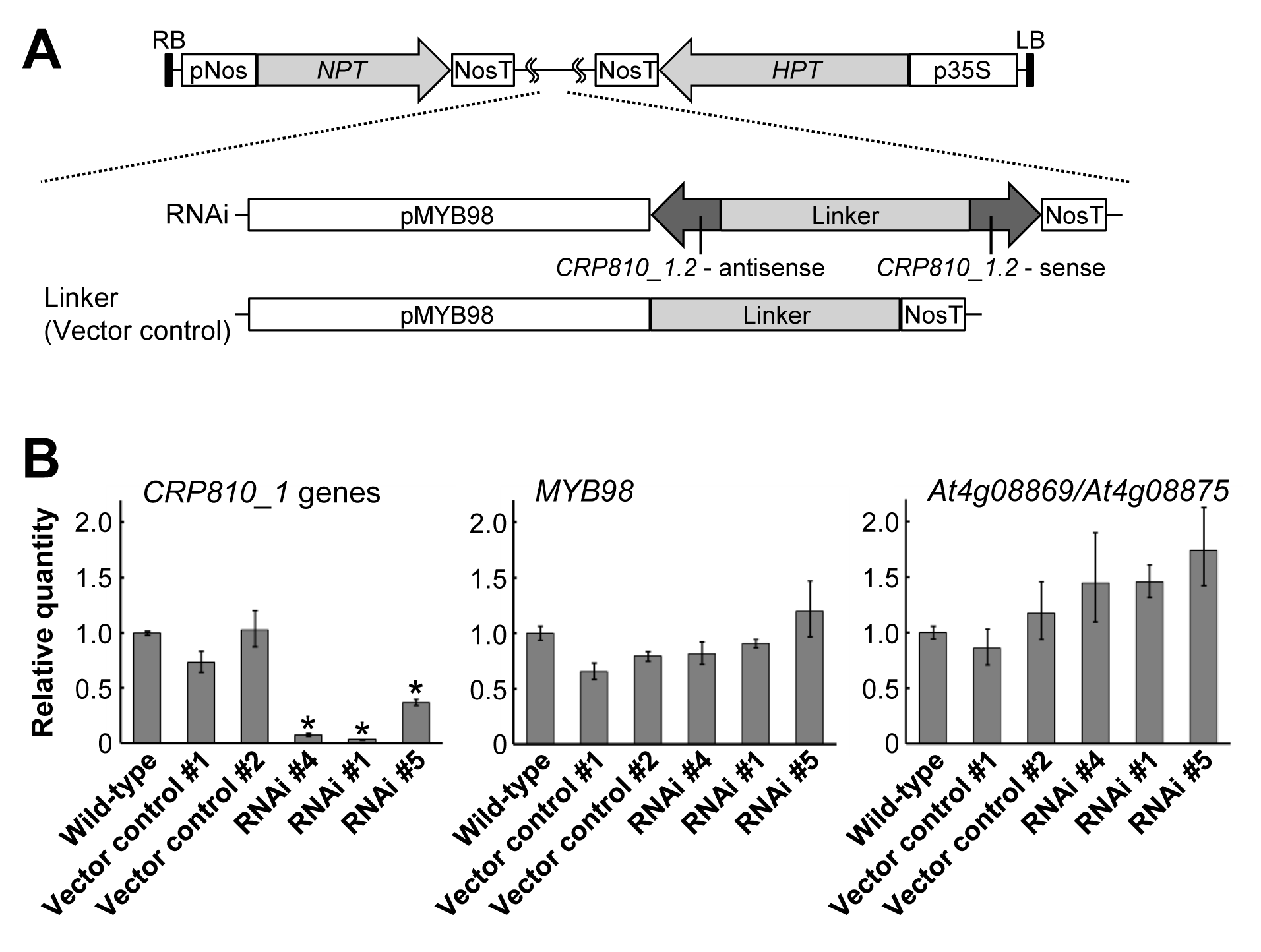

Supplement: Figure S4 — Knockdown analysis of CRP810_1 genes by RNAi, related to Figure 4 . (A) Schematic of the RNAi constructs. The upper graphic represents the T-DNA region between the right (RB) and left (LB) borders. The genes encoding neomycin phosphotransferase (NPT) and hygromycin phosphotransferase (HPT) confer resistance to kanamycin and hygromycin, respectively. The middle and lower graphics represent the structures of CRP810_1-RNAi (RNAi) and the linker (vector control). The MYB98 promoter (pMYB98) was used to drive the RNAi sequence in the synergid cells. The linker sequence is nucleotides 72–1,067 of the GUS coding sequence. CRP810_1.2-antisense and -sense sequences can form dsRNA when the sequence is transcribed. pNos, nopaline synthase promoter; NosT, nopaline synthase terminator; p35S, cauliflower mosaic virus (CaMV) 35S promoter. (B) Real-time qRT-PCR analysis of CRP810_1, MYB98, and At4g08869/At4g08875 in the pistils of the RNAi and vector control lines. To confirm the specific downregulation of the CRP810_1 genes, synergid-specific MYB98 and the closest CRP810 genes (At4g08869 and At4g08875) were used. The relative quantities are the expression levels relative to that in the wild type. Two independent vector control lines and three independent RNAi lines were thought to be homozygous T3 lines because the siblings of each T3 plant were drug-resistant. Each expression level was normalized to that of ACT2. The data are the means and standard errors of three sibling plants. Asterisks indicate significant differences compared with the wild type according to Dunnett's test (p<0.05) by ANOVA. (TIF) [file pbio.1001449.s004.tif]

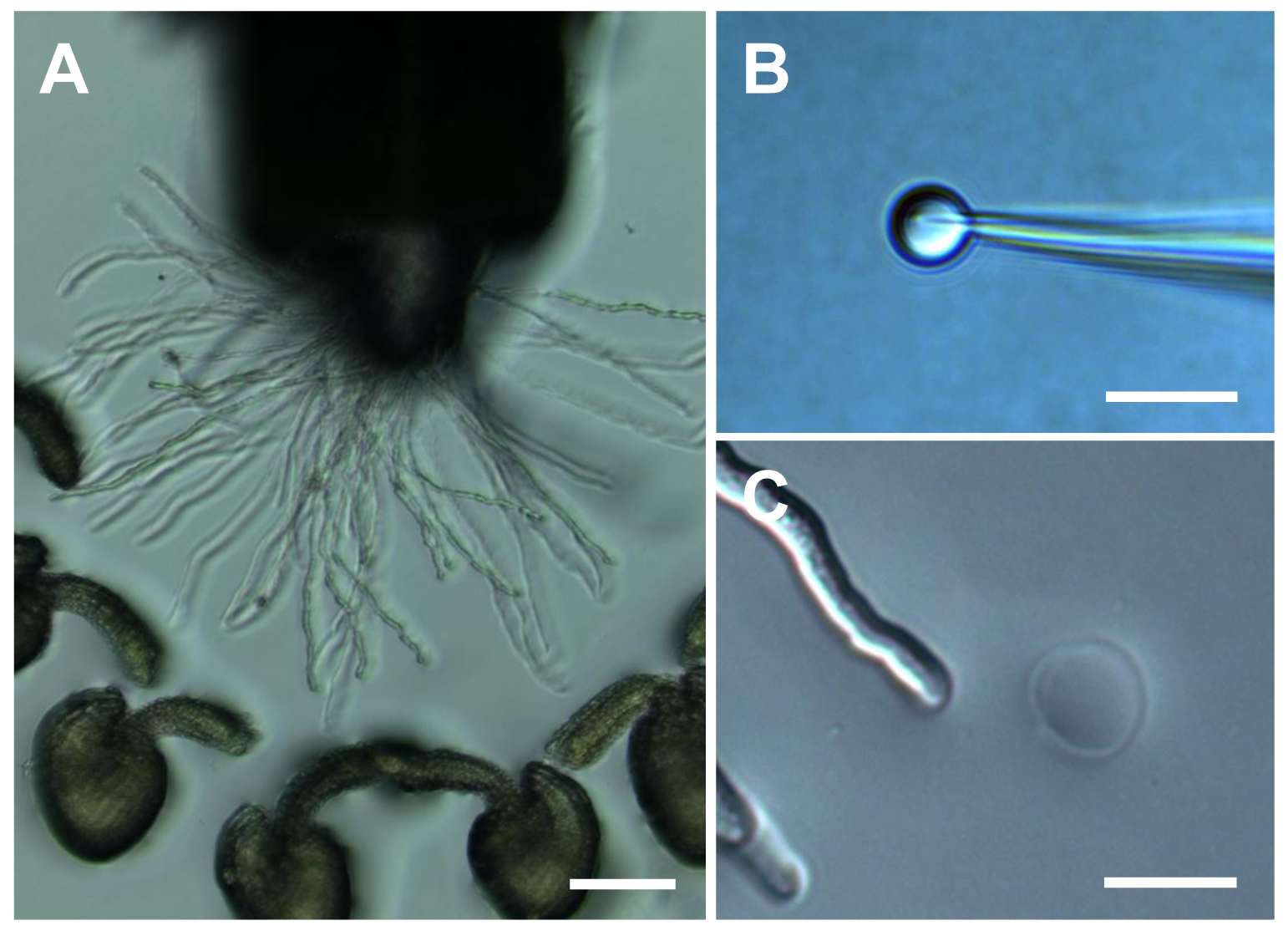

Supplement: Figure S5 — In vitro pollen tube attraction assay of Arabidopsis , related to Figures 5 and 6 . (A) In vitro pollen tube growth. Pollen tubes through the cut style were grown on medium using an in vitro system. Ovules were co-cultured because unknown protein(s) derived from the ovules could promote pollen tube growth and/or attraction. Around 5 h after the start of incubation, the pollen tube attraction assays were started. The picture shows pollen tubes emerging from the cut style and ovules 6 h after the start of incubation. Scale bar, 100 µm. (B) A gelatin bead manipulated with a glass needle. The gelatin bead was attached to the tip of the glass needle and placed on the medium through micromanipulation. Scale bar, 20 µm. (C) A gelatin bead placed in front of the tip of a pollen tube. The gelatin bead was slowly dissolved in the medium to spread the proteins embedded in it. Scale bar, 20 µm. (TIF) [file pbio.1001449.s005.tif]

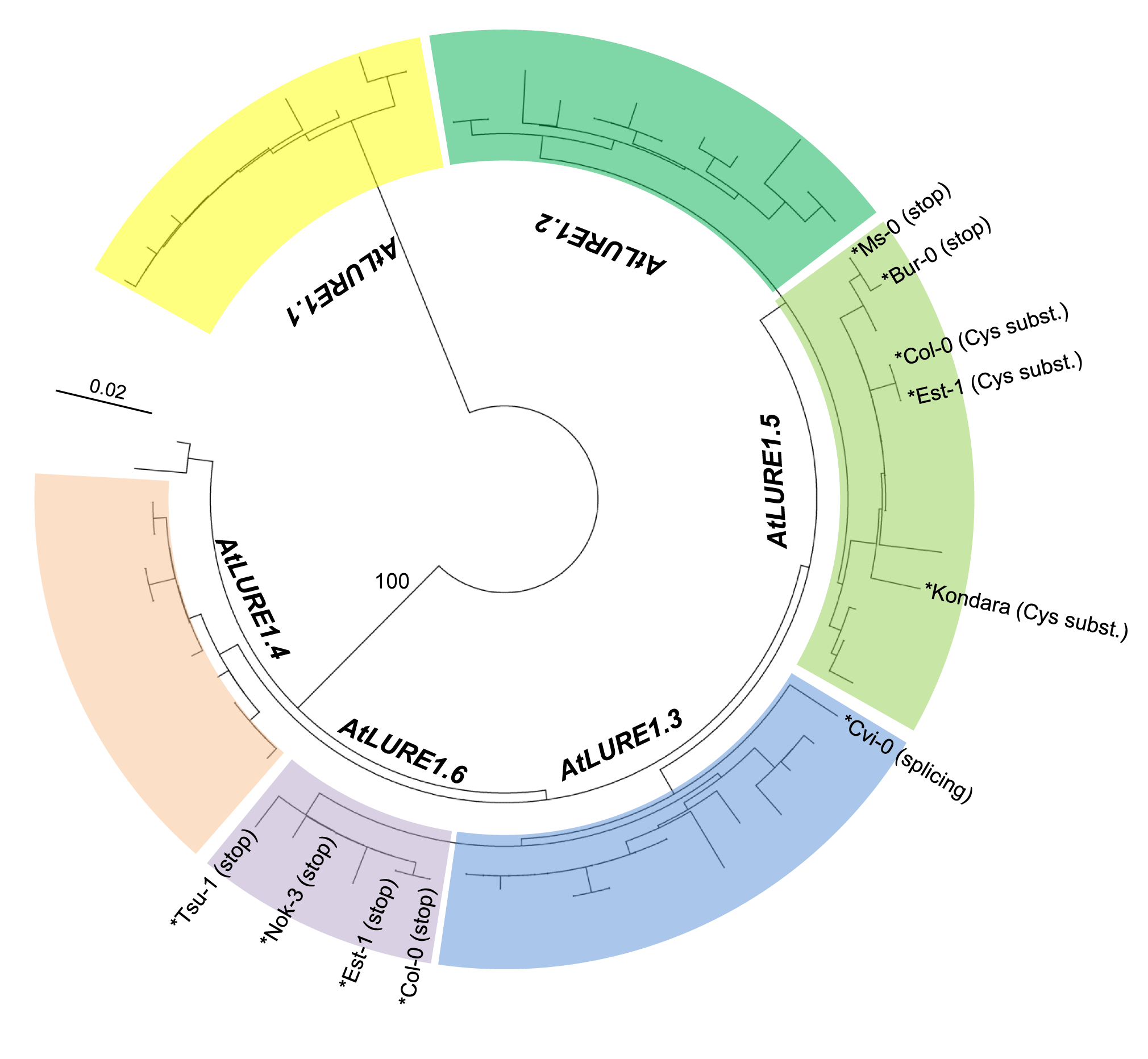

Supplement: Figure S6 — Sequence variation of AtLURE1 ( CRP810_1 ) genes in various accessions of A. thaliana . A phylogenetic tree of six AtLURE1 (AtLURE1.1 to 1.6) from the Col-0 accession and 60 sequences from 12 other A. thaliana accessions based on the coding region of their genomic sequences. Each background color indicates subtrees containing AtLURE1.1 to 1.6. The scale shows the number of substitutions per site. The genes with asterisks are probably nonfunctional. Also see Table S2. (TIF) [file pbio.1001449.s006.tif]

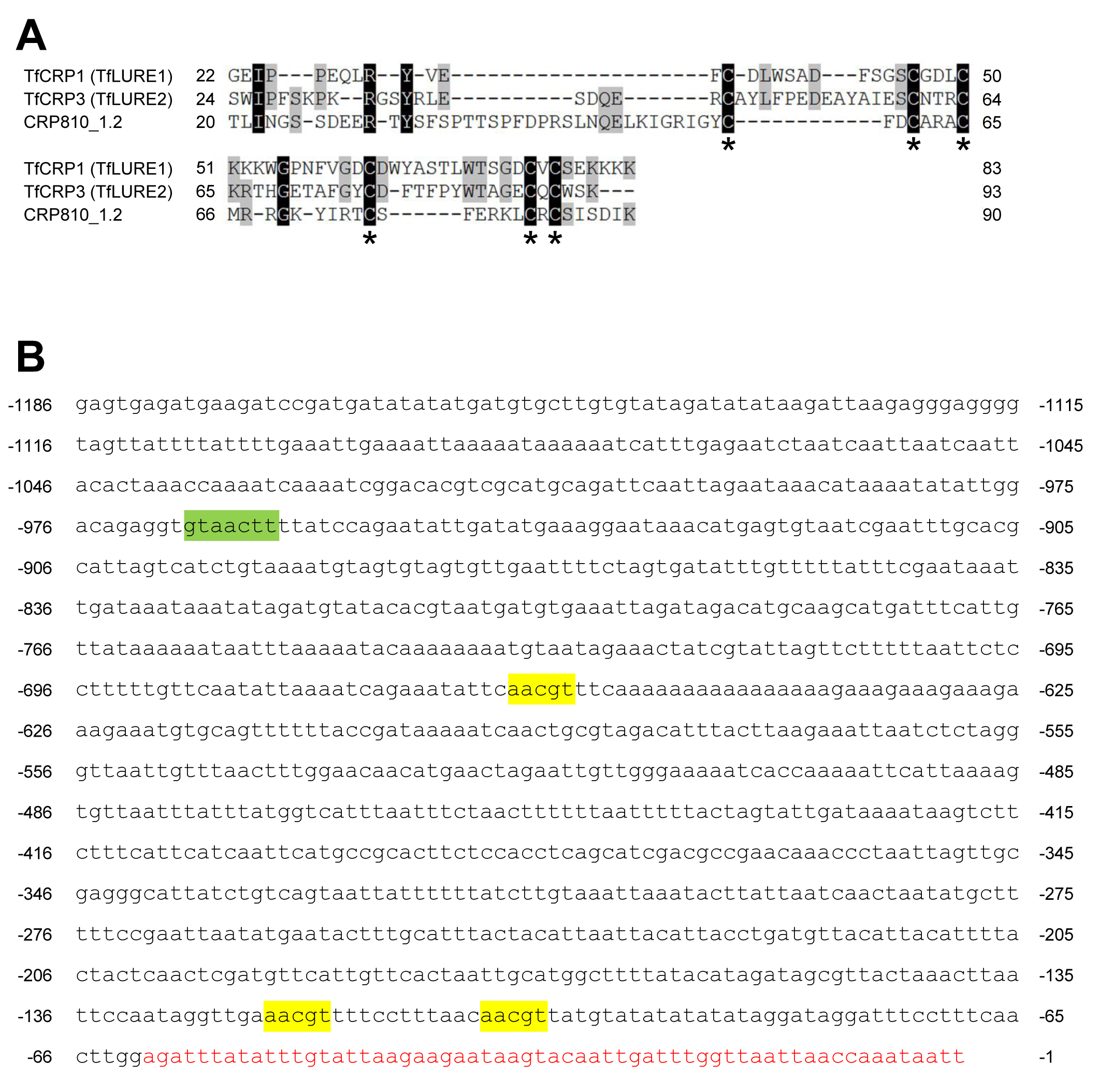

Supplement: Figure S7 — Sequence comparison of TfLUREs and CRP810_1.2 peptides and the promoter sequence of TfLURE2 . (A) Putative mature peptides of TfCRP1 (TfLURE1) and TfCRP3 (TfLURE2), pollen tube attractants of T. fournieri, and CRP810_1.2 (AtLURE1.2) as a representative CRP810_1 peptide are shown. Asterisks mark conserved cysteine residues. (B) Upstream sequence of a translation start site of TfLURE2. A green highlight and yellow highlights indicate cis-element GTAACNT, which is suggested to be MYB98-binding sequence, and cis-element AACGT, which is necessary and sufficient for AtLURE1.2 (DD2) expression in the synergid cell of A. thaliana, respectively [23]. Red letters mark 5′ untranslated region of TfLURE2. (TIF) [file pbio.1001449.s007.tif]

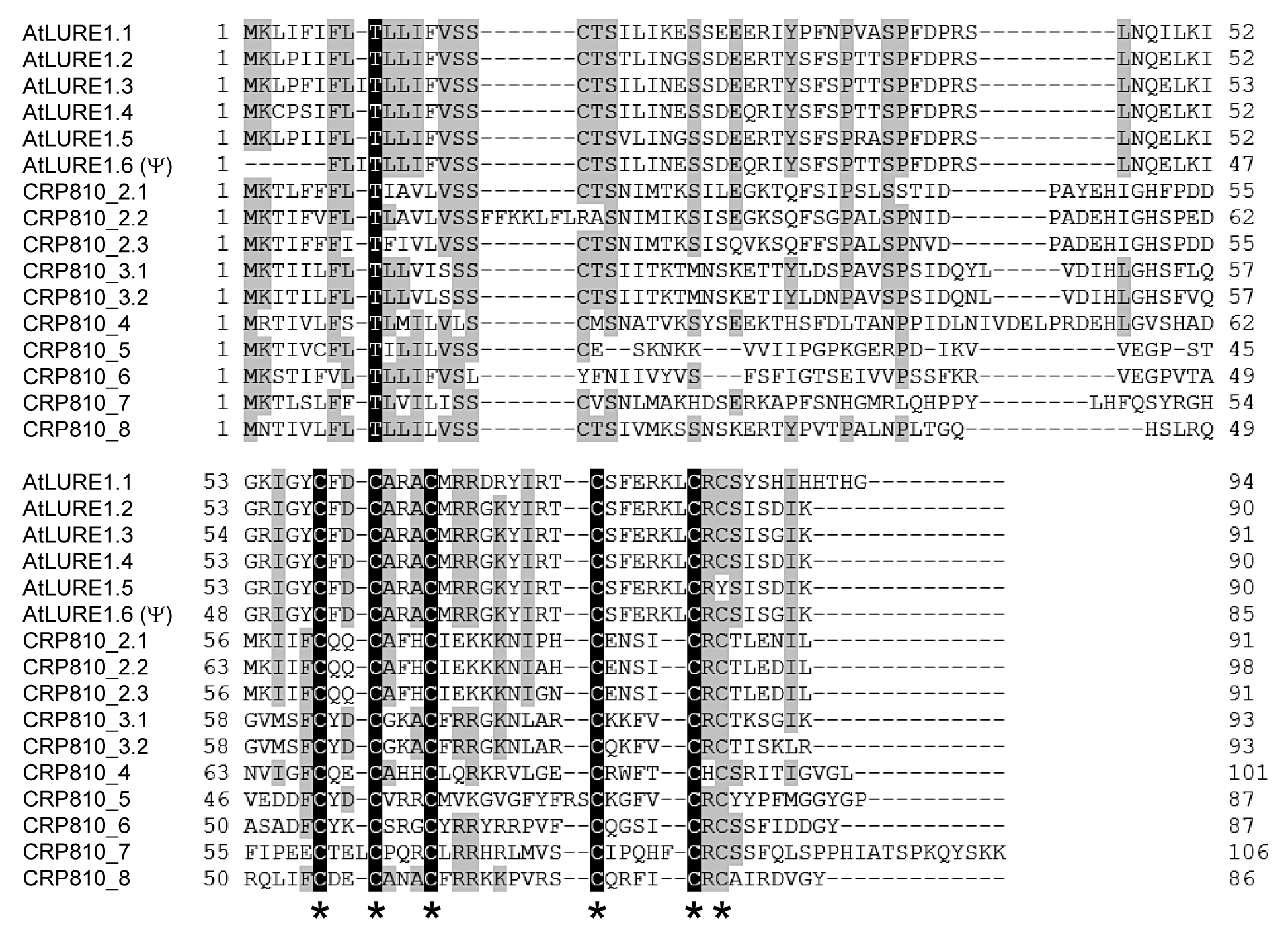

Supplement: Figure S8 — A multiple alignment of CRP810 peptides. A multiple alignment of the full-length amino acid sequences of 16 CRP810 peptides in A. thaliana. Black and gray backgrounds indicate amino acids conserved among 16 and ≥eight sequences, respectively. Asterisks mark conserved cysteine residues. The accession numbers of the CRP810 genes are as follows: CRP810_2.1 (At5g48515), _2.2 (At5g48595), _2.3 (At5g48605), _3.1 (At4g08869), _3.2 (At4g08875), _4 (At5g50423), _5 (At5g18403), _6 (At5g18407), _7 (At5g60805), and _8 (At4g08485). (TIF) [file pbio.1001449.s008.tif]

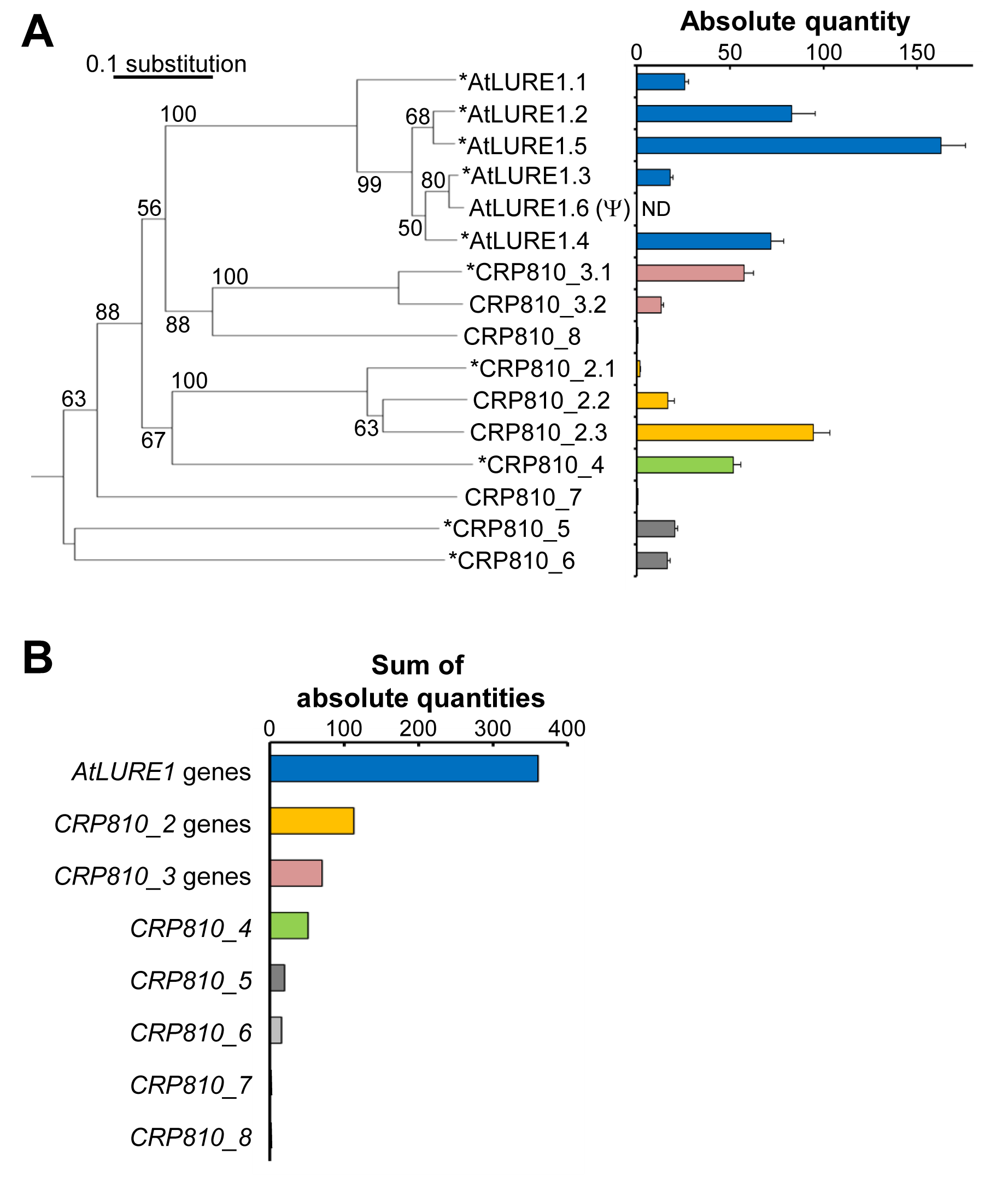

Supplement: Figure S9 — Expression analysis of CRP810 genes in A. thaliana . (A) A phylogenetic relationship (left) and absolute gene expression levels in the pistil (right) of the CRP810 genes. The tree is a portion of a phylogenetic tree of 317 DEFL peptides. Asterisks mark genes downregulated in myb98 according to a genome-wide analysis [21]. The bootstrap values more than 50 for the neighbor-joining (NJ) method are indicated as percentages. The scale shows the number of amino acid substitutions per site. Absolute quantity represents the copy number of cDNA per that of MYB98 cDNA. The means and standard deviations of three independent experiments are shown. (B) Absolute gene expression levels for paralogous gene groups. For AtLURE1 (CRP810_1), CRP810_2, and _3, the values are the sum of the absolute quantities for each paralogous gene. The CRP810 genes were numbered from CRP810_1 (AtLURE1) to _8 according to the level of expression in each group. (TIF) [file pbio.1001449.s009.tif]
