# Supplementary material for: A Species-Specific Cluster of Defensin-Like Genes Encodes Diffusible Pollen Tube Attractants in Arabidopsis
Source: PLoS Biol. 2012 Dec 18;10(12):e1001449. doi: 10.1371/journal.pbio.1001449 (PMC3525529; doi:10.1371/journal.pbio.1001449)
Supplement: Text S1 — Supporting methods and references. (DOC) [file pbio.1001449.s015.doc]

**Supporting Methods**

**Plant Growth Conditions**

*Arabidopsis thaliana* seeds were sterilized and germinated on plates containing 0.5× Murashige and Skoog salts (Wako Pure Chemical Industries), 1% sucrose, 0.5× Gamborg’s vitamin solution (Sigma), and 0.3% gelrite (Wako Pure Chemical Industries). Ten-day-old seedlings were transferred to soil and grown under continuous light at 22°C. To select *maa3/MAA3* heterozygous mutants, semi-sterility was checked. To select *myb98/MYB98* and *ccg/CCG* heterozygous mutants, T-DNA insertion was confirmed by PCR using the T-DNA-specific primer LBa1 (5’-TGGTTCACGTAGTGGGCCATCG-3’) and genomic sequence primer for *MYB98* (5’-TGGGTATAGTAAAAACACACATTAAATGTC-3’) or *CCG* (5’-ATTTGAGGGATCATCTCCTGACG-3’). Growth condition for *Arabidopsis lyrata* was similar with the exception of a vernalizing cold treatment. For the treatment, *A. lyrata* plants in the rosette stage were grown in 8 h photoperiod at 4°C for at least 1 month.

**Sequence Determination of *AtLURE1* Orthologs in *A*. *lyrata***

Orthologous *DEFL* genes in *A. lyrata* were identified by a BLAST search at the Department of Energy Joint Genome Institute (JGI) (http://genome.jgi-psf.org/Araly1/Araly1.home.html). Four *CRP810_1* orthologs (*AlCRP810_1.4*, *1.7*, *1.9*, and *1.10*) were found by BLASTp (BLAST protein vs. protein). Additionally, six orthologs were found by BLASTn (BLAST nucleotide vs. nucleotide); *AlCRP810_1.1*, *1.2*, *1.3*, *1.5*, and *1.6* were found in a region adjacent to *AlCRP810_1.4* and *1.7* on scaffold_8, while *AlCRP810_1.8* was found on scaffold_97, which showed no synteny to the *A. thaliana* genome. *AlCRP810_1.9* and *1.10* were tandemly duplicated genes within 2 kb on scaffold_1021, which also showed no synteny to the *A. thaliana* genome. These 10 genes were named according to their locations (Table S1). Other orthologous *DEFL* genes in *A. lyrata* were found by BLASTn (Table S1).

Synteny analysis of genomic regions containing *CRP810_1* genes, *CRP700* (*ATTI*) genes, *CRP580* (*LCR*) genes, and *CRP860* (*SCRL*) genes was performed using genomic data from the two species. For genomic regions containing *AlCRP810_1* genes, one gap on scaffold_8 (4535001-4570000) and two gaps on scaffold_8 (4222301-4251300) were sequenced using PCR-based direct sequencing. The reactions were performed using three pairs of primers: 5’-GTTTAAAAGAATTGATTAGGTCACC-3’ and 5’-GGTATTGTAATCATTTTAAAAAAATTC-3’, 5’-TCCGAAACCCGTAGACAGACAC-3’ and 5’-GCGGTCACCATCTTTCATC-3’, and 5’-AAGACTCTTATCACCTTCAAGGTC-3’ and 5’-AACCGAAACATTGGCTTTAGAG-3’, respectively. The products were sequenced using the same primers and sequencing primers for the latter two products, 5’-GGTTTTGTGAGAGTGCAGTTG-3’ and 5’-CAGTTTGAGGAGATGTACCAGAG-3’, and 5’-AAAGGATGTAGCATCTAAACACAG-3’, 5’-CCAAAAAACACATTATTTAGGGAG-3’, and 5’-CTTGGAGACGATCGACGATG-3’, respectively. The gap-filled sequences confirmed that no *AlCRP810_1* gene existed in these regions. The region containing *At5g43285* (*CRP810_1.1* or *AtLURE1.1*) showed synteny to the region including *AlLURE1.1* to *1.7*. On the other hand, no *AlLURE1* gene was found in a syntenic region containing *At5g43510* (*CRP810_1.2* or *AtLURE1.2*) to *At5g43525* (*CRP810_1.5* or *AtLURE1.5*). These two syntenic regions were drawn with reference to sequences from *A*. *thaliana* chromosome 5 (17360001–17386000) and *A*. *lyrata* scaffold_8 (4535001–4570000), and sequences from *A*. *thaliana* chromosome 5 (17469401–17495400) and *A*. *lyrata* scaffold_8 (4222301–4251300), respectively (Figure 2B). In a similar way, syntenic regions for *CRP700* (*ATTI*) genes, *CRP580* (*LCR*) genes, and *CRP860* (*SCRL*) genes were drawn with reference to sequences from *A*. *thaliana* chromosome 2 (18062001–18076000) and *A*. *lyrata* scaffold_4 (21267001–21289000), sequences from *A*. *thaliana* chromosome 4 (14423001–14440000) and *A*. *lyrata* scaffold_7 (5359000–5340001), and sequences from *A*. *thaliana* chromosome 1 (22448001–22466000) and *A*. *lyrata* scaffold_2 (2822000–2794001), respectively (Figure S1B).

**Quantitative Real-Time RT-PCR**

For quantitative real-time PCR using Power SYBR Green PCR Master Mix (Applied Biosystems), the PCR mixture consisted of 0.5 µM primers, appropriately diluted cDNA, and 1× master mix in a volume of 15 µl. The PCR program consisted of 95°C for 10 min, followed by 45 cycles at 95°C for 15 s and 60°C for 1 min. Subsequently, the melting curve was plotted to check the specificity of amplification according to the default setting. The C_T_ of each sample was determined according to the average of two or three technical replicates.

To quantify the absolute expression levels of the *CRP810_1* genes and *MYB98*, the standard curve method was applied using template vectors of known copy number as standards. The vectors were constructed using a Zero Blunt TOPO PCR Cloning Kit (Invitrogen) to clone PCR products of the target sequences, which were amplified from pistil cDNA. To amplify each of the paralogous *CRP810_1* genes, amplification refractory mutation system (ARMS) PCR analysis [1] was applied. For ARMS PCR analysis, primers with one or two mismatched nucleotides immediately upstream of the SNP site in the target sequence were designed. Specific amplification of a single gene was confirmed by real-time PCR using vectors of non-targeted paralogous genes as templates. The means and standard deviations of the absolute expression levels for each gene were calculated from the values in three independent pistil cDNA samples and normalized to the *MYB98* absolute expression level.

To quantify the relative expression levels, the comparative C_T_ (ΔΔC_T_) method was applied as described below. First, for each sample, the ΔC_T_ of each gene compared to an internal control gene, *ACT2* (*At3g18780*), was determined using the formula ΔC_T_ = C_T_ (gene of interest) – C_T_ (*ACT2*). Next, for each tissue or genotype, the mean ΔC_T_ and standard error was calculated. Finally, the expression levels as the relative quantity (RQ) to a reference sample were calculated using the formula RQ = 2 – (mean of ΔCt [sample of interest] – mean of ΔCt [reference sample]).

**Constructs for Promoter GFP Analysis**

To prepare a subcloning vector containing the *GFP* sequence, *GFP* and the 5’ linker sequence, amplified from pGWB4 [2] using the primers 5’linker_F+SpeI-SmaI (5’-gtaactagttctcccgggAAGGGTGGGCG-3’) and GFP_R+SacI (5’-taggagctctactcgagattggtaccCTTGTACAGCTCGT-3’), were introduced into pT7Blue (Novagen) using *Spe*I and *Sac*I. Next, the promoter sequence and promoter sequences plus coding regions without the stop codon of each *CRP810_1* (*AtLURE1*) gene were amplified with iProof High-Fidelity DNA Polymerase (Bio-Rad) from genomic DNA. The amplified products were subcloned into the GFP vector using *Hin*dIII and *Sma*I. *GFP*-fused sequences were cut from the subcloned vectors and transferred to the pGWB500 series vector [3] with *Hin*dIII and *Sac*I, resulting in the binary vectors pGWB500-*pCRP810_1::GFP* series and pGWB500-*pCRP810_1::CRP810_1-GFP* series. These constructs were transformed into wild-type *A*. *thaliana* plants (Col-0) using the floral dip method. Transformed plants were selected on medium containing 20 mg/l hygromycin B (Wako Pure Chemical Industries).

**Immunostaining**

IgGs in the pre-immune serum and anti-CRP810_1.2 serum were purified by affinity chromatography (HiTrap Protein G HP, GE Healthcare). For immunostaining, the carpel walls were removed from the pistil, resulting in ovules on the septum. The ovules were then fixed using 4% paraformaldehyde in PBS for 40 min or a 9:1 mixture of ethanol and acetic acid overnight. After treatment with 0.2% Triton X-100 for 5 min and blocking with 3% bovine serum albumin (BSA) for 1 h at 37°C, the samples were treated with purified pre-immune and anti-CRP810_1.2 antibodies (1:1000 dilution) in 1% BSA for 1 h at 37°C. The samples were then treated with Alexa Fluor 488-conjugated anti-rabbit goat IgG (1:1000 dilution; Invitrogen) for 1 h at 37°C. The stained ovules were observed by epifluorescence and confocal laser-scanning microscopy.

**Purification of Recombinant Peptides and In Vitro Attraction Assay**

The procedures used (i.e., cloning into the expression vector and purification of the His-tagged peptides) are described in the main text. The mutated CRP810_1.5-Y84C sequence was generated by PCR using the primer CRP810_1.5 MP_F+BamHI and mismatched primer (5’-TTATTTAATATCACTAATACTGcAACGAC-3’), and a second round of PCR using the same primer pair as for CRP810_1.5.

Following expression and purification of the His-tagged peptides by metal affinity chromatography, the procedures were similar to those described previously for TfLUREs [4]. The peptides were dialyzed (Spectra/Por3 MWCO:3500; Spectrum Laboratories) and refolded using a solution containing glutathione (reduced and oxidized forms; Wako Pure Chemical Industries) and l-arginine ethyl ester dihydrochloride (Sigma) for 4 days at 4°C. For the in vitro attraction assay using gelatin beads, 1 µl of purified peptide in buffer (50 mM Tris-HCl, pH 7.0) was mixed with 2 µl of 10% (w/v) gelatin (Nacalai Tesque) solution and 1 µl of 1 mM Alexa Fluor 488 or 568 conjugated with 10-kDa dextran (Invitrogen). Gelatin beads were formed by adding 200 µl of hydrated silicone oil, vortexing, and cooling on ice. The gelatin beads were manipulated using the point of a glass needle and placed in front of the tip of a pollen tube under an inverted microscope (IX71, Olympus). Pollen tubes growing toward the beads with a >30º change were designated as “attracted” pollen tubes.

For the in vitro attraction assay, pollen tube growth medium [5,6] was used. A total of 120–150 µl of medium was poured into the well, which was made from silicone rubber with a 10 mm × 36 mm rectangular hole on a cover glass. A pistil emasculated 1 day before was hand-pollinated and cut off at the junction between the style and ovary using a 27-gauge needle. Next, the cut stigma with the style was placed on the medium; the cut edge of the style was at a right angle to the medium. The ovules were also placed on the side of the cut edge from which pollen tubes emerged through the style. After setting the style and ovules, another cover glass was covered with silicone rubber to keep the medium from drying out during incubation. The style and ovules on the medium were incubated at 22°C in the dark until the pollen tube attraction assay was performed.

**Generation of RNAi Constructs**

The *CRP810_1.2* (*AtLURE1.2*) nucleotide sequence was used as a representative trigger for the inhibition of *CRP810_1* genes by RNAi since it shares >96% identity with *CRP810_1.3*, *1.4*, and *1.5*, and 89% identity with *CRP810_1.1*. Furthermore, it contains a common sequence of up to 33 bp with *CRP810_1.1* to *1.5*. Since RNAi can be mediated by RNAs 21 and 22 bp in length [7], the RNAi construct against *CRP810_1.2* was expected to target all *CRP810_1* genes. The RNAi construct was designed to express double-stranded RNA (dsRNA) under control of the synergid-specific *MYB98* promoter (Figure S4A). To generate inverted repeat sequences of *CRP810_1.2* coding sequence for the RNAi construct, nucleotides 72–1067 of the *GUS* coding sequence as a linker for the dsRNA, which was amplified with GoTaq DNA polymerase (Promega) using the primers GUSlinker_F (5’-TCGCGAAAACTGTGGAATTG-3’) and GUSlinker_R (5’-CCGACAGCAGCAGTTTCATC-3’), was first cloned into pT7Blue T-vector (Novagen), resulting in the *GUS-linker* sequence. *CRP810_1.2* was also amplified using the primers *CRP810_1.2*_F+BamHI-SpeI (5’-cgcggatccactagtATGAAGTTGCCTATT-3’) and *CRP810_1.2*_R+XbaI-EcoRI (5’-ccggaattctctagaTTATTTAATATCACT-3’). The amplified product was ligated into a flanking region of the *GUS-linker* sequence using *Spe*I and *Xba*I, and then ligated into another flanking region using *Bam*HI and *Eco*RI, resulting in *CRP810_1-RNAi*. The *MYB98* promoter, which specifically drives the RNAi sequence in synergid cells, was amplified using the primers pMYB98_TOPO_F (5’-caccGGTGAAGAGAGAGAGAGAGAGATTG-3’) and pMYB98_R+EcoRI-SphI (5’-gcatgcgaattcTGTTTTGGAAAGGAG-3’), and introduced into pENTR/D-TOPO (Invitrogen). The *CRP810_1-RNAi* sequence and *GUS-linker* sequence were connected downstream of the *MYB98* promoter using *Eco*RI and *Sph*I. The *pMYB98:: CRP810_1-RNAi* and *pMYB98::GUS-linker* were transferred to the binary vector pGWB1 [2] using the LR recombination reaction (Invitrogen), resulting in the binary vectors pGWB1- *CRP810_1-RNAi* (RNAi) and pGWB1-*GUS-linker* (vector control). These constructs were transformed into wild-type *A*. *thaliana* plants by the floral dip method. Transformed plants were selected with 50 mg/l hygromycin B (Wako Pure Chemical Industries).

**Analysis of Pollen Tube Guidance in the Pistil**

For the analysis of pollen tube guidance in the pistil, the carpel walls were removed from the pistil about 1 day after flowering using a 27-gauge needle, and the resulting ovules and septum were stained with 0.1% aniline blue in 0.1 M K_3_PO_4_ without fixation. To evaluate abnormal pollen tube guidance around the micropyle, the number of ovules was counted only when the pollen tube(s) was visible from the base of the funiculus to the micropylar opening of the ovule in the prepared slide. Otherwise, the ovule was designated ND. Because a pollen tube was observed at almost all funiculi in the RNAi pistil and wild type, this criterion for the count appeared to have no bias. Defects in micropylar pollen tube guidance were classified into two groups. A class I abnormality was defined as one or more “wandering” pollen tubes with ultimate entry of the tube into the micropyle. A class II abnormality was defined as no pollen tubes entering the micropyle after growing up to the funiculus. “Wandering” was defined as a pollen tube that took a 180º turn back on the funiculus or grew on the surface of the ovule.

**Sequence Determination of *AtLURE1.1* to *1.6* in Various Accessions**

In 12 accessions of *A*. *thaliana* (Cvi-0, Est-1, Mr-0, Tsu-1, Nok-3, Fei-0, Ts-1, Pro-0, Kondara, Ms-0, Bur-0, and Ws-2), the genomic sequences of *AtLURE1.1* to *1.6* were investigated by PCR and direct sequencing using the Sanger method. These 12 accessions were picked out according to the genetic relationships among 95 accessions [8]. They were likely to be distributed throughout the genetic relationships.

The coding regions of the genomic sequences were determined. First, PCR to amplify the coding region was performed using genomic DNA from each accession. The primers were designed using SNPs from the *AtLURE1.1* to *1.6* sequences in Col-0. Second, nested PCR was performed for *AtLURE1.1* to *1.6*. Primary PCR primers were designed for the sequences of a flanking gene or intergenic region. Secondary PCR primers were designed to amplify all of the *AtLURE1* genes in Col-0. The products were subsequently sequenced. If multiple peaks were detected, the sequence could not be determined. Sequencing after cloning was avoided because the nucleotides, especially in the heteroduplex DNA, may have been modified by *E*. *coli*.

**Supporting References**

1. Bai RK, Wong LJC (2004) Detection and quantification of heteroplasmic mutant mitochondrial DNA by real-time amplification refractory mutation system quantitative PCR analysis: a single-step approach. Clin Chem 50: 996-1001.
2. Nakagawa T, Kurose T, Hino T, Tanaka K, Kawamukai M, et al. (2007) Development of series of Gateway Binary Vectors, pGWBs, for realizing efficient construction of fusion genes for plant transformation. J Biosci Bioeng 104: 34-41.
3. Nakagawa T, Suzuki T, Murata S, Nakamura S, Hino T, et al. (2007) Improved Gateway Binary Vectors: high-performance vectors for creation of fusion constructs in transgenic analysis of plants. Biosci Biotechnol Biochem 71: 2095-2100.
4. Okuda S, Tsutsui H, Shiina K, Sprunck S, Takeuchi H, et al. (2009) Defensin-like polypeptide LUREs are pollen tube attractants secreted from synergid cells. Nature 458: 357-361.
5. Palanivelu R, Preuss D (2006) Distinct short-range ovule signals attract or repel *Arabidopsis thaliana* pollen tubes *in vitro*. BMC Plant Biol 6: 7.
6. Hamamura Y, Saito C, Awai C, Kurihara D, Miyawaki A, et al. (2011) Live-cell imaging reveals the dynamics of two sperm cells during double fertilization in *Arabidopsis thaliana.* Curr Biol 21: 497-502.
7. Elbashir SM, Lendeckel W, Tuschl T (2001) RNA interference is mediated by 21- and 22-nucleotide RNAs. Genes Dev 15: 188-200.
8. Aranzana MJ, Kim S, Zhao K, Bakker E, Horton M, et al. (2005) Genome-wide association mapping in *Arabidopsis* identifies previously known flowering time and pathogen resistance genes. PLoS Genet 1: 531-539.
